# Phenotypic CRISPR screens identify NLRX1 as an essential activator of the human mitochondrial permeability transition

**DOI:** 10.1073/pnas.2535298123

**Published:** 2026-02-25

**Authors:** William C. Valinsky, Robert P. Ray, Kathy S. Schaefer, Jonathan B. Grimm, Carla Nicolini, Luke D. Lavis, David E. Clapham

**Affiliations:** ^a^Janelia Research Campus, HHMI, Ashburn, VA 20147

**Keywords:** mitochondria, permeability transition, NLRX1, calcium, MCU

## Abstract

The mitochondrial permeability transition (mPT) is an evolutionarily conserved destructive process that permeabilizes the inner mitochondrial membrane in response to calcium overload. The molecular mechanism underlying the mPT is not established. To unambiguously identify essential proteins, we designed two phenotypic assays for mitochondrial calcium overload and applied them to FACS-based CRISPR screening in human cells, ultimately evaluating 19,113 genes. The first screen studied mitochondrial membrane potential (MMP) collapse in response to calcium overload. Top-ranked genes were the essential proteins of the mitochondrial calcium uniporter complex, MCU and EMRE, reflecting that the calcium-induced MMP collapse results from mitochondrial calcium entry and not the mPT. The second screen measured the permeability of the inner mitochondrial membrane. Here, the fluorescent interaction of a membrane impermeant ~600 Da dye and a mitochondrial-targeted HaloTag protein was studied under mPT activating conditions; calcium overload and the thiol-reactive molecule phenylarsine oxide. With secondary validation, we identified four protein-encoding genes that delayed or prevented the mPT under knockout: NF2, REST, BPTF, and NRLX1. Knockout of the nonmitochondrial proteins BPTF, NF2, or REST increased mitochondrial calcium retention capacity (CRC). However, calcium release or sensitivity to cyclosporin A (CsA) persisted, indicative of mPT sensitizers. Only knockout of the mitochondrial matrix protein, NLRX1, increased CRC, abolished calcium release, and was CsA-insensitive. This top-ranked hit of the mitochondrial permeability screen meets the definition of an essential mPT activator. Integral membrane proteins, including all previously proposed mPT candidates, were not essential activators.

## Significance

Mitochondria utilize calcium to increase ATP synthesis. However, excessive matrix calcium activates the mitochondrial permeability transition (mPT), a process that permeabilizes the mitochondrial inner membrane and leads to cell death. While initially characterized 50 y ago, the proteins underlying the process are unclear, although integral membrane proteins were expected to be the porous entities during calcium overload. Here, we designed two assays to study the mPT using high-throughput methodologies. By surveying 19,113 proteins in human cells, we identified four proteins that sensitize the human mPT, but only one that was essential for mPT activation, mitochondrial-localized NRLX1. Surprisingly, NLRX1 is not an integral membrane protein, and our work did not identify any essential integral membrane proteins for the human mPT.

Mitochondria are endosymbiotic organelles (1, 2) that synthesize most cellular ATP by the proton-pumping chemiosmotic mechanism (3–6). Proton pumping across the inner mitochondrial membrane (IMM) generates an electronegative potential of approximately −140 mV to −180 mV (7–11). Mitochondria typically express a Ca^2+^-selective inwardly rectifying ion channel (12), the mitochondrial Ca^2+^ uniporter complex (13–16), which mediates rapid Ca^2+^ entry down the electrochemical gradient. Mitochondria buffer cytoplasmic Ca^2+^ (17, 18) and matrix Ca^2+^ increases the activity of citric acid cycle dehydrogenases (19–22) to increase ATP production (23).

First demonstrated in rat kidney homogenates (24) and mitochondria (25–28), high [Ca^2+^] (roughly >200 nmol/mg) is irreversibly destructive to mitochondrial oxidative phosphorylation. Absorbance-based measurements revealed that high [Ca^2+^] decreased mitochondrial light scattering, inferred as swelling (29–34), and later supported by electron microscopy (35). In addition, high [Ca^2+^] uncoupled respiration and abolished ^45^Ca^2+^ accumulation (34, 35). These structural and functional observations became known as the mitochondrial permeability transition (mPT) (36–39), further described as a Ca^2+^-induced increase of IMM permeability to polyethylene glycol (PEG) molecules up to ~1,500 Da (37). Several properties were characterized in the founding studies, including indistinguishable Ca^2+^-release and structural transformation kinetics (39), mPT occurrence in a de-energized state (38), and the existence of hypotonically swollen mitochondria without an activated mPT (36). Proton uncoupler-induced Ca^2+^ release could not be fully explained by the mPT (39), and its causes remain controversial (40–44).

Mitochondrial Ca^2+^ accumulation is correlated with ischemia-reperfusion (IR) injury (45–50). Ca^2+^ chelators (46) and Ca^2+^ channel antagonists (45, 51, 52) were protective against IR damage, but Ca^2+^ alone does not mediate all the consequences of IR injury. During prolonged ischemia (60 min), reoxygenation (reperfusion) increased lipid peroxidation and decreased cellular thiol content (53) due to oxygen toxicity (54). Reactive oxygen species (ROS) also caused Ca^2+^ release from liver mitochondria (55, 56), likely via the mPT (57). However, oxidative stress is considered an agonist for the Ca^2+^-dependent mPT (57, 58).

These findings led to the identification of a small molecule inhibitor of the mPT, cyclosporin A (CsA). CsA prevented Ca^2+^-related mitochondrial dysfunction (59) by slowing or preventing the mPT (60–62), thereby protecting against IR injury (63). CsA antagonizes cyclophilin (64), a peptidyl-prolyl *cis-trans* isomerase (65). The mitochondrial matrix expresses one cyclophilin (62, 66–69), PPIF. Mice with PPIF knockout (KO) were more resistant to IR injury (70, 71), and the Ca^2+^ threshold for mPT activation was increased, but the mPT was not prevented (70–72). PPIF KO, however, is a complex phenotype in which varied ischemia durations lead to improved or worsened outcomes in mice (73). CsA given prior to reperfusion failed to improve cardiac outcomes in clinical trials (74, 75).

Mitochondrial integral membrane proteins have been proposed as mPT pore (mPTP) components. These include the adenosine nucleotide translocases (ANTs) (76, 77), the mitochondrial phosphate carrier (PiC) (78), the voltage-dependent anion channel (VDAC) (79, 80), and F_0_F_1_ ATP synthase (81–84). However, independent studies challenged these claims, as mitochondrial swelling or Ca^2+^ release persisted in KO cells (85–91). Essential mPT proteins remain unknown, although they are unlikely to arise from mtDNA-encoded genes (92) or the outer mitochondrial membrane (OMM) (93). Here, we sought to identify the essential proteins of the mPT in an unbiased manner in human cells. We developed two mitochondrial Ca^2+^ overload assays, each with a mPT phenotypic fluorescent reporter and each amenable to unbiased genome-wide CRISPR-Cas9 screening with Fluorescence Activated Cell Sorting (FACS). We further assessed genetic hits by mitochondrial Ca^2+^ release and CsA-sensitivity.

## Results

### A Scalable Phenotypic Assay for Mitochondrial Ca^2+^ Overload.

HAP1, an immortalized human cell line with a near-haploid karyotype (94), was selected for assay development. HAP1 mitochondria have classical mPT characteristics, including Ca^2+^ release, absorbance-based swelling, molecular size limits, and pharmacology (89–91). HAP1 cells are also ideal for genetic screening efforts (95, 96), as gene disruption efficiency is expected to be high in its mostly haploid karyotype. As expected, digitonin-permeabilized (97) HAP1 cells demonstrated repetitive Ca^2+^ uptake spikes in the Ca^2+^ retention capacity (CRC) assay until a large, coordinated release of Ca^2+^ occurred, interpreted as the mPT (*SI Appendix*, Fig. S1*A*). HAP1 cells also exhibited an uncoupler-induced Ca^2+^ release (UCR) after subthreshold mPT Ca^2+^ loading (*SI Appendix*, Fig. S1*B*).

**Fig. 1.**
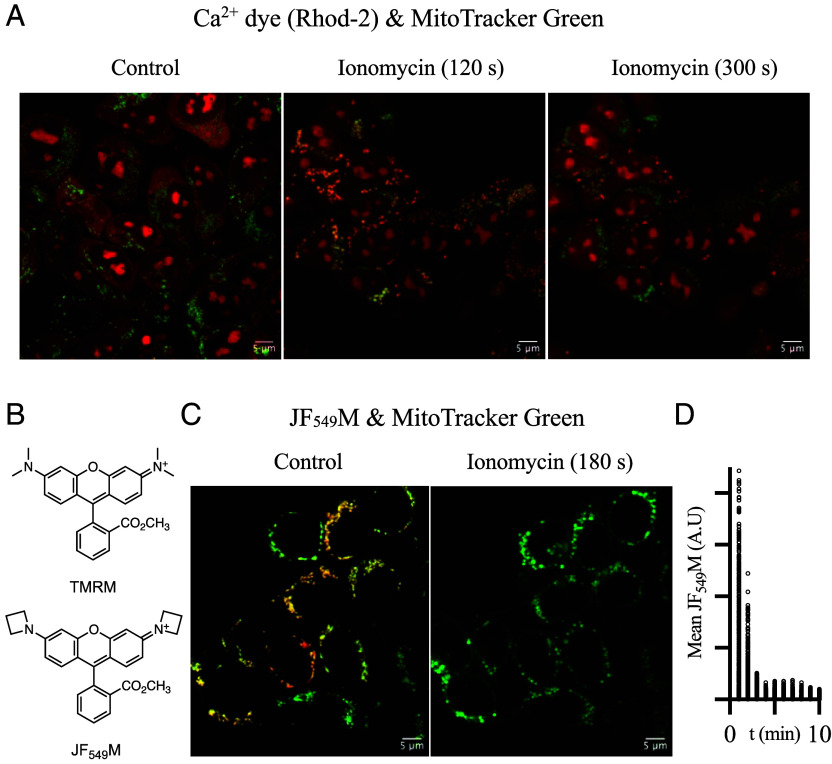
A scalable phenotypic assay for mitochondrial Ca^2+^ overload. (*A*) Ionomycin transiently increases matrix Ca^2+^. Confocal images (63X) of intact WT HAP1 cells incubated with the Ca^2+^ indicator, 5 μM Rhod-2, AM, and the mitochondrial reporter, 100 nM MitoTracker Green. Images were captured in control (*Left*), 120 s after 10 μM ionomycin application (*Middle*), and 300 s after 10 μM ionomycin application (*Right*). Images were collected in a time series experiment. (Scale bar, 5 μm.) (*B*) Chemical structures of rhodamine-based mitochondrial voltage dyes TMRM (*Top*) and JF_549_M (*Bottom*). (*C*) Ionomycin induces mitochondrial membrane potential (MMP) collapse. Intact HAP1 cells were incubated with 25 nM JF_549_M and 100 nM MitoTracker Green. Confocal images were captured before (*Left*) and 180 s after (*Right*) 10 μM ionomycin application. (Scale bar, 5 μm.) (*D*) Ionomycin-induced JF_549_M signal depletion occurs in minutes. The fluorescence of mitochondrial particles, defined by size and circularity parameters using MitoTracker Green, was tracked over time from the full frame image of (*C*) using the multimeasure function of ImageJ.

For intact cells, mitochondrial Ca^2+^ levels were monitored using Rhod-2, AM (5 μM), a Ca^2+^-activated dye that accumulates in mitochondria (98, 99). Based on colocalization with the mitochondrial marker MitoTracker Green (100 nM), Rhod-2 exhibited minimal mitochondrial signal in unstimulated conditions (Fig. 1 *A*, *Left*), indicating HAP1 mitochondria (at rest) have matrix [Ca^2+^] below ~500 to 700 nM, the Rhod-2 Ca^2+^ K_D_ (100, 101). With the nuclear stain Hoechst 33342 (1 μg/mL), a bright nuclear Rhod-2 signal was also observed (*SI Appendix*, Fig. S2). To raise intracellular [Ca^2+^], we applied the Ca^2+^ ionophore ionomycin (102) (10 μM). Rhod-2 and MitoTracker Green rapidly colocalized (Fig. 1 *A*, *Middle*; orange structures). By 5 min, the Rhod-2 signal diminished and the remaining structures appeared punctate (Fig. 1 *A*, *Right*; green structures). While these were indicators of mitochondrial Ca^2+^ overload and mPT activation, the nuclear Rhod-2 signal and transient mitochondrial signal rendered Rhod-2 inadequate for large-scale screening.

In dual simultaneous imaging CRC experiments, Ca^2+^ release was correlated with absorbance-based swelling in mouse mitochondria (86, 103, 104) and MMP collapse in human mitochondria (105, 106). Thus, we hypothesized that MMP collapse may be a reporter for the human mPT. To maximize the signal, a new, brighter, rhodamine-based MMP dye was designed. This dye combined the methyl ester group of Tetramethylrhodamine, Methyl Ester (TMRM) with the Janelia Fluor 549 scaffold (107) (Fig. 1*B*). The resultant chemical, JF_549_M, showed mitochondrial specificity (*SI Appendix*, Fig. S3 *A*-*C*) and was depleted by MMP depolarization with the uncoupler carbonyl cyanide m-chlorophenyl hydrazone (CCCP; 5 μM) (*SI Appendix*, Fig. S3 *D* and *E*). By flow cytometry and with or without CCCP, JF_549_M (25 nM) was consistently brighter than TMRM (25 nM), suggesting an unchanged dynamic range (*SI Appendix*, Fig. S3*F*).

For subsequent assay development, we exclusively used the brighter JF_549_M as the MMP dye. To evaluate if MMP could be a reporter for our live cell mitochondrial Ca^2+^ overload assay, HAP1 cells were incubated with 25 nM JF_549_M and 100 nM MitoTracker Green and then treated with 10 μM ionomycin. Within minutes of ionomycin application, JF_549_M fluorescence rapidly declined and did not recover, indicative of MMP collapse (Fig. 1 *C* and *D*).

### Genome-Wide CRISPR-Cas9 FACS Screen With the Mitochondrial Membrane Potential (MMP) Assay.

**Fig. 2.**
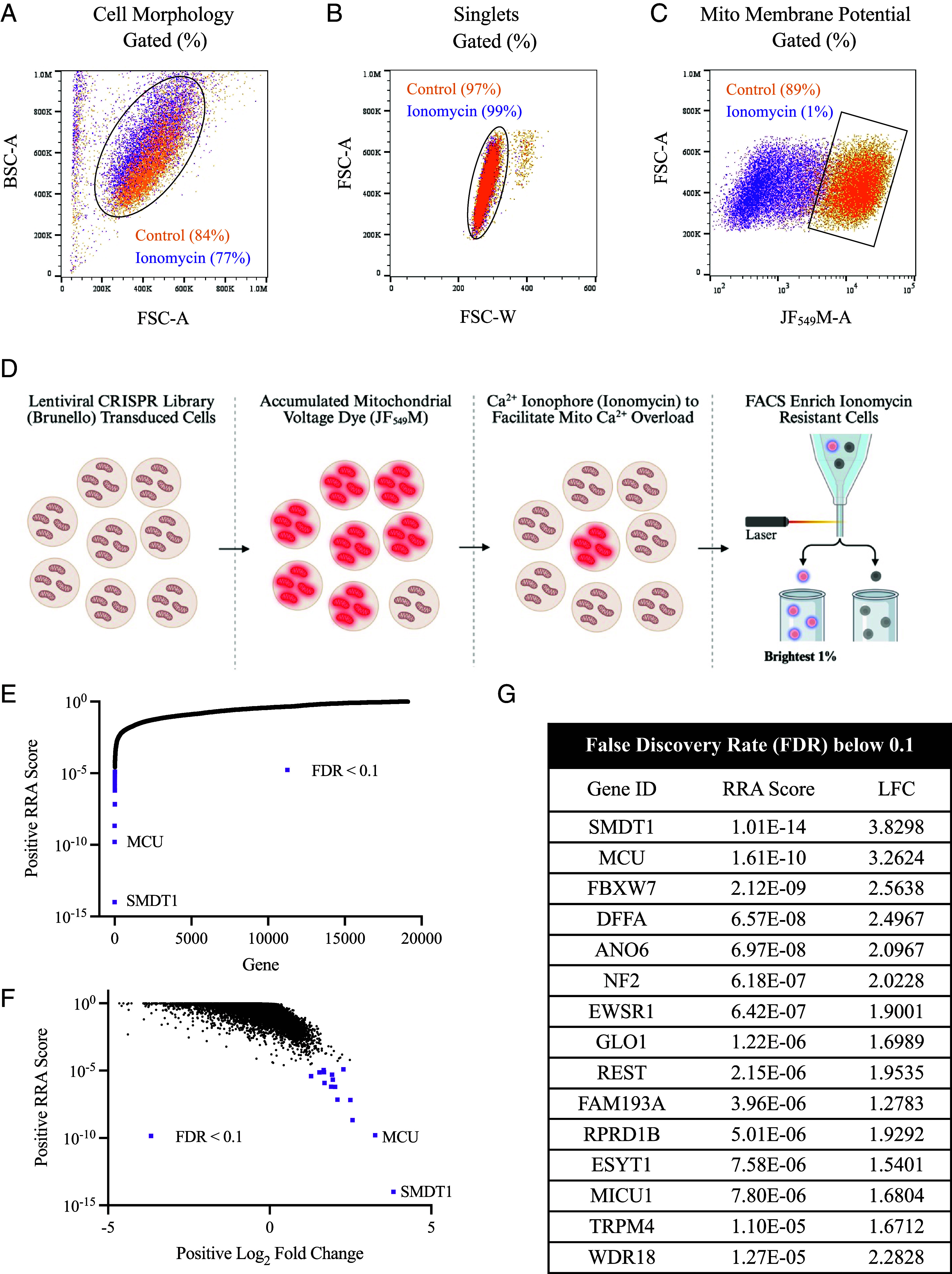
Genome-wide CRISPR-Cas9 FACS screen for the mitochondrial membrane potential (MMP) assay. (*A*–*C*) Ionomycin depolarizes mitochondria without widespread cell death. Flow cytometry plots of intact WT HAP1 cells incubated with 25 nM JF_549_M (15 min, 37 °C), then with (purple) or without (orange) 10 μM ionomycin (5 min, 37 °C). (*A*) Living cells were gated within the cell morphology (BSC-A vs. FSC-A) plot. (*B*) singlets, derived from the living cells gate, were represented by the gated population in the FSC-A vs. FSC-W plot. (*C*) MMP, derived from the singlet plot, was evaluated by JF_549_M brightness. In all plots, parentheses indicate the percentage gated. (*D*) Schematic of the genome-wide CRISPR-Cas9 screen. HAP1 cells were transduced with the Brunello library at low multiplicity of infection (MOI). The MMP dye (25 nM JF_549_M) was applied (15 min, 37 °C), followed by the Ca^2+^ ionophore (10 μM ionomycin; 5 min, 37 °C), then washout. The brightest 1% of the JF_549_M population (ionomycin-resistant cells) were FACS collected for positive sgRNA enrichment analysis. (*E*–*G*) Statistical enrichment analysis by MAGeCK RRA. Plots of Positive RRA Score vs Gene (*E*) and Positive RRA Score vs. Positive Log_2_ Fold Change (*F*). Statistically enriched genes were marked by FDR < 0.1 (purple), and the top two hits, SMDT1 and MCU, are labeled. Table (*G*) of all statistically enriched hits (FDR < 0.1) ranked by Positive RRA Score.

The MMP assay was optimized for cell sorting conditions. By flow cytometry, a 5 min incubation of 10 μM ionomycin (followed by a prompt washout) did not cause large-scale cell death (Fig. 2*A*) or cell agglutination (Fig. 2*B*). However, ionomycin depolarized the MMP, as gated singlets from the control JF_549_M population declined from ~89% to ~1% (Fig. 2*C*). This methodology was applied to a genome-wide CRISPR-Cas9 screen with the Brunello library (108), where KOs that protected against the ionomycin-induced MMP collapse (JF_549_M depletion) were FACS enriched (Fig. 2*D*). Approximately 38 million events were recorded and ~305,000 ionomycin-resistant particles were collected, representing the brightest ~0.8% of treated input cells.

sgRNAs were amplified from the population and sequenced using next-generation sequencing (NGS). Data were analyzed by the MAGeCK Robust Rank Algorithm (RRA) (109), and quality control metrics were within bounds. In control samples, zero sgRNA counts were 182 (99.8% coverage), the Gini index (measure of diversity) was 0.094, and mapped read depth was 256. In total, 77,259 sgRNAs were assessed, representing 19,113 genes (Dataset S1). Candidates were ranked by positive RRA score, and statistically enriched genes were defined by a false discovery rate (FDR) < 0.1 (110) and log_2_ fold change (LFC) > 1.0. Only 15 candidates met the statistical cut-off (Fig. 2 *E*–*G*).

### Mitochondrial Ca^2+^ Import Primarily Causes MMP Collapse.

We evaluated the positive RRA hits by Gene Set Enrichment Analysis (GSEA) using the Comprehensive Resource of Mammalian Protein Complexes (CORUM; Protein Complexes), Gene Ontology (GO) Biological Processes, the Kyoto Encyclopedia of Genes and Genomes (KEGG), and the Reactome (111). Positively enriched pathways that met statistical thresholds (-log_10_FDR > 1.3; FDR < 0.05) were the mitochondrial Ca^2+^ uniporter complex (Protein Complexes), Ca^2+^ import into the mitochondrion (GO Biological Process), and processing of SMDT1 (Reactome Pathways) (*SI Appendix*, Fig. S4). This is supported by individual examination of 15 hits, where three genes of the mitochondrial Ca^2+^ uniporter complex were found: SMDT1 (EMRE) (16, 112), MCU (13, 15, 112), and MICU1 (15, 16), ranked 1st, 2nd, and 13th, respectively (Dataset S1 and Fig. 2*G*).

GSEA identified the importance of rapid mitochondrial Ca^2+^ influx to our phenotypic assay; however, it did not provide insight into Ca^2+^ release mechanisms. To study Ca^2+^ release and the mPT, each candidate was genetically disrupted in HAP1 cells and studied by CRC and UCR. HAP1 KOs were obtained from Horizon Biosciences or produced internally, with frameshift indel mutations verified by Sanger sequencing for internally produced cells (*SI Appendix*, Fig. S5). WDR18, the 15th enriched candidate, failed KO attempts, suggesting a requirement of this gene for HAP1 cell propagation.

**Fig. 3.**
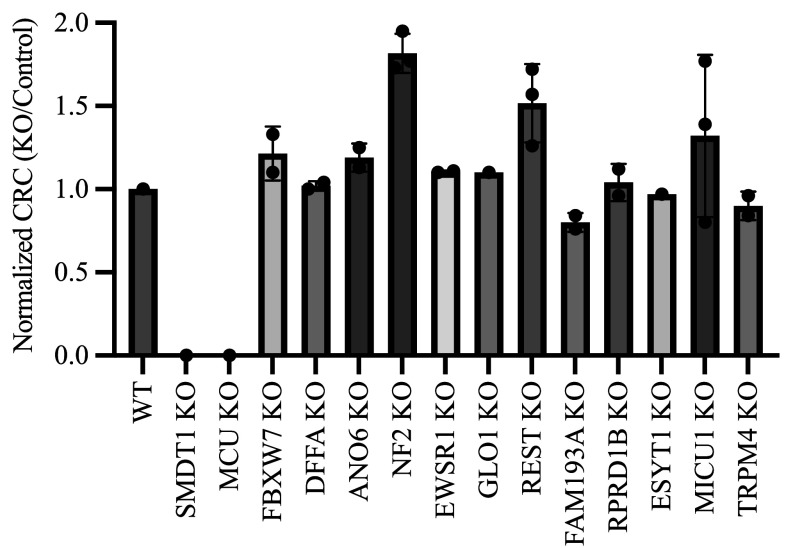
Mitochondrial Ca^2+^ release is not prevented by knockout (KO) of any hit from the MMP CRISPR screen. Plot of normalized (KO/control) Ca^2+^ uptake spikes before Ca^2+^ release in the CRC assay. Each technical triplicate from a single experiment was averaged and divided by the paired control. KOs which increased Ca^2+^ uptake count by ~25% (>~1.25) were repeated (n = 3). Data are mean ± S.D. In all datasets, digitonin-permeabilized HAP1 cells were seeded at 2 million cells/well in a 96-well blackout plate for Ca^2+^ imaging (1 μM Fura-FF; F340/F380).

SMDT1 KO and MCU KO prevented Ca^2+^ uptake, and thus there was no Ca^2+^ release in CRC or UCR (*SI Appendix*, Fig. S6 *A* and *C*). These hits were not considered mPT candidates due to the persistence of mPT phenotypic outputs in MCU KO mitochondria under ionophore-induced Ca^2+^ uptake (113). The remaining hits were studied by CRC and UCR to evaluate if they were essential for the mPT. By CRC, hits were assessed by either increased pulse count before Ca^2+^ release (the mPT) or by prevention of Ca^2+^ release. In NF2 KO and REST KO, CRC pulse counts were increased, but large Ca^2+^ release events still occurred (*SI Appendix*, Fig. S6*B*). Overall, NF2 KO tolerated more Ca^2+^ pulses before Ca^2+^ release than REST KO. All other hits did not consistently increase CRC (Fig. 3). UCR was not prevented in any KO; however, several KOs delayed UCR (*SI Appendix*, Fig. S6 *D*–*H*), including NF2 KO and REST KO (*SI Appendix*, Fig. S6*D*). Several KOs altered Ca^2+^ uptake (*SI Appendix*, Fig. S6 *D*–*H*), with MICU1 KO having the largest effect (*SI Appendix*, Fig. S6*E*).

### A High-Throughput Assay for the mPT.

To directly study the mPT, a new assay was developed which satisfied the original definition set forth by Haworth and Hunter in 1979: the Ca^2+^-induced membrane transition in mitochondria (37–39). In this definition, the mPT is an increase of IMM permeability to sucrose, nicotinamide adenine dinucleotide phosphate (NADP)^+^, and PEG molecules up to ~1,500 Da in response to Ca^2+^ overload.

**Fig. 4.**
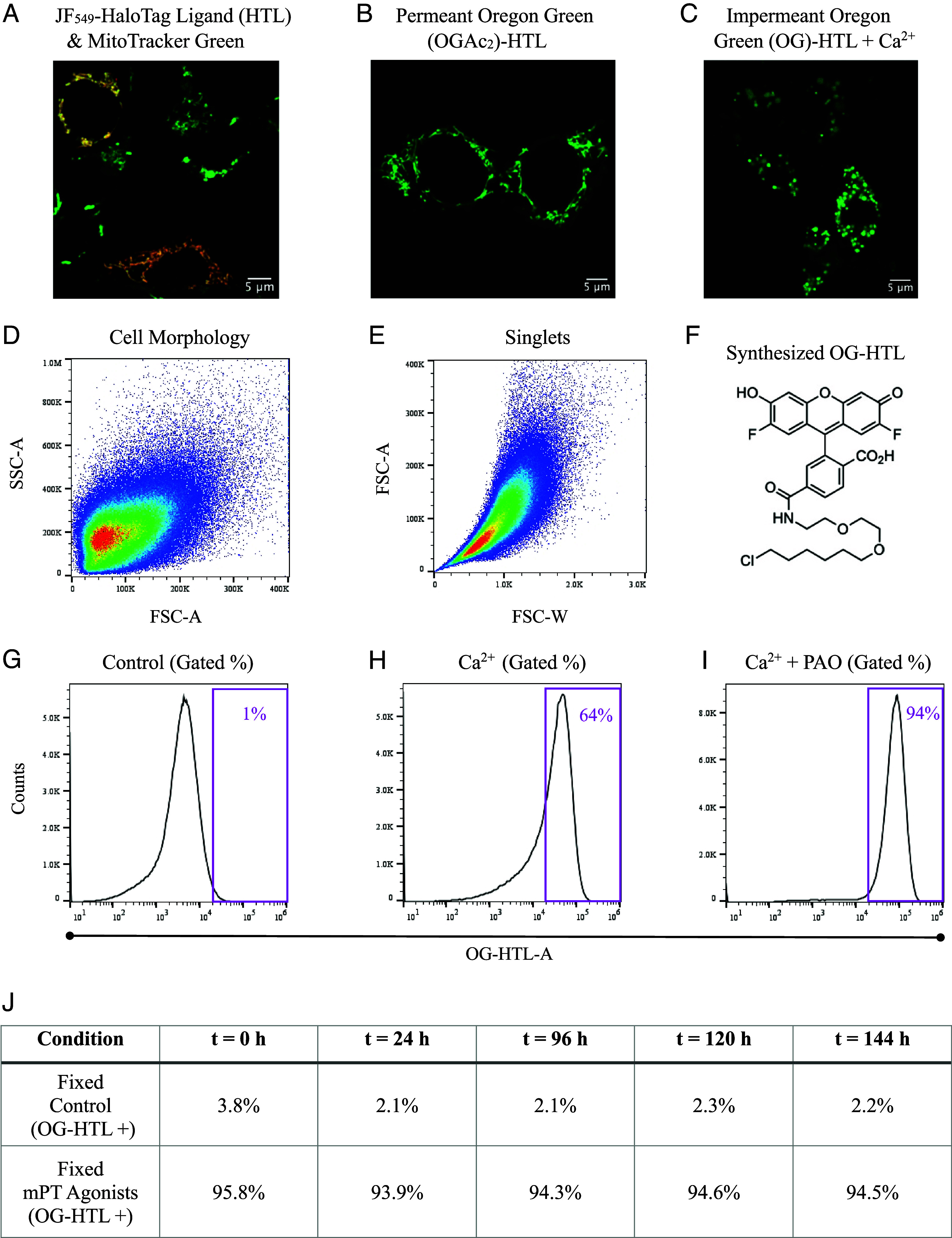
High-throughput assay of the mitochondrial permeability transition (mPT). (*A*) HaloMTS is specifically expressed in mitochondria. Confocal image (63X) of intact WT HAP1 cells transiently expressing HaloMTS and stained with 100 nM JF_549_-HaloTag ligand and 100 nM MitoTracker Green. The merged image shows colocalization in orange or yellow structures. (Scale bar, 5 μm.) (*B*) Confocal image (63X) of intact HAP1 cells transiently expressing HaloMTS and stained with membrane permeant 30 nM acetylated Oregon Green HaloTag ligand (OGAc_2_-HTL). (Scale bar, 5 μm.) (*C*) Membrane impermeant Oregon Green HaloTag ligand stains punctate mitochondria in high free Ca^2+^. Confocal image (63X) of digitonin-permeabilized HAP1 cells transiently expressing HaloMTS and stained with 30 nM deacetylated (impermeant) Oregon Green HaloTag ligand (OG-HTL) with 400 μM free external [Ca^2+^]. (Scale bar, 5 μm.) (*D* and *E*) Flow cytometry plots of cell morphology (SSC-A vs. FSC-A; *D*) and derived singlets (FSC-A vs. FSC-W; E) in digitonin-permeabilized HAP1SR-HaloMTS cells. (*F*) Chemical structure of synthesized impermeant Oregon Green HaloTag ligand (OG-HTL). (*G*–*I*) Ca^2+^ and thiol-reactive PAO are required to maximally activate the mPT. Flow cytometry histograms comparing 30 nM OG-HTL (impermeant) staining of digitonin-permeabilized HAP1SR-HaloMTS cells in the following conditions: control (*G*), 400 μM Ca^2+^ (*H*), and 400 μM Ca^2+^ + 20 μM PAO (*I*). Particles were first gated for cell morphology (SSC-A vs. FSC-A), followed by singlets (FSC-A vs. FSC-W), then OG-HTL fluorescence (OG-HTL-A). The OG-HTL-A gate corresponds to ~1% of bright cells in control conditions. (*J*) 24 h after fixation, OG-HTL fluorescence is stable for 5 d. Digitonin-permeabilized HAP1SR-HaloMTS cells were fixed (4% PFA, 30 min on ice), washed, and analyzed in a cell sorter with conditions and gates as in (*G* and *I*). Daily recordings for control (*Top*) and mPT agonists (*Bottom*) were performed. OG-HTL positive particles are listed as percentages of the total population.

We designed a receptor–ligand fluorescent assay, amenable to mitochondrial Ca^2+^ overload, that conforms to the size limits of the mPT while having minimal background. We used the HaloTag protein, which binds its ligand (dye-conjugated) via a specific, fast, and irreversible reaction (114). HAP1 cells were transiently transfected with HaloMTS (115), a HaloTag protein containing an N-terminal mitochondrial localization sequence. By comparison to MitoTracker Green (100 nM) in confocal microscopy, JF_549_-HaloTag ligand (JF_549_-HTL; 100 nM) exclusively stained mitochondrial structures (Fig. 4*A*).

For the mPT, the HaloTag ligand dye must be membrane impermeant, yet IMM permeant in mPT conditions. As impermeant versions of JF_549_-HaloTag ligand are larger than NADP^+^ (>744 Da), we used the smaller Oregon Green HaloTag ligand (OG-HTL; ~600 Da). Commercially available OG-HTL (30 nM, Promega G2801) did not alter staining patterns in HaloMTS expressing HAP1 cells (Fig. 4*B*). However, it is membrane permeant (acetylated; OGAc_2_-HTL) and not appropriate for mPT studies. In preliminary experiments, OGAc_2_-HTL was deacetylated in cell media (30 nM, OG-HTL) to confer membrane impermeability (116), and verified as impermeant by the absence of intracellular fluorescence in intact cells. To provide access to mitochondria, the plasma membrane was permeabilized using 50 ng/μL digitonin (97). With a nominally Ca^2+^-free (2 mM EGTA) solution, no mitochondrial labeling was observed. However, with 400 μM free [Ca^2+^], swollen green puncta emerged (Fig. 4*C*), likely reporting mPT activation.

This assay identifies resistance to Ca^2+^ overload by the cells lacking fluorescence (dark). Contamination by nonexpressing cells is one fallibility. Consequently, we designed a system to reduce false positives. First, HaloMTS was stably expressed under a UCOE-SFFV promoter to minimize transgene silencing (117). Second, HAP1 cells screened for haploid status (HAP1 Screening Ready; HAP1SR cells) were acquired to improve KO efficiency. The resultant stably expressing HAP1SR-HaloMTS cells were FACS-enriched twice. The final cell line stained >99% positive for OGAc_2_-HTL (intact cells) and did not diminish over 8 further passages (*SI Appendix*, Fig. S7 *A*–*C*), within the boundaries of the genome-wide screen (Passage 7, P7).

In adapting the mitochondrial permeability assay for cell sorting, we observed that digitonin permeabilization impacted cell morphology and singlet plots. By cell morphology, the population was small (FSC-A; Fig. 4*D*) and showed low complexity (SSC-A; Fig. 4*D*) compared to intact cells (Fig. 2*A*). Singlet plots were also shaped as tear drops (Fig. 4*E*) instead of ovals (Fig. 2*B*). No obvious second population was present in either permeabilized plot. Therefore, >95% of particles in each plot were captured.

To prepare for a large-scale experiment, we synthesized the impermeant OG-HTL (618 Da; Fig. 4*F*). Several mPT-stimulating parameters were tested to maximize OG-HTL labeling, including [Ca^2+^], [Mg^2+^], incubation temperature, incubation time, and pH (37). However, by flow cytometry, OG-HTL labeling saturated at ~64% of singlets compared to ~1% of matched controls (Fig. 4 *G* and *H*). In permeabilized *Drosophila melanogaster* cells, mitochondrial swelling and small molecule release did not occur at the limit of endogenous Ca^2+^ uptake (118, 119). Coapplication of the potent mPT agonist, phenylarsine oxide (PAO) (72, 120–124), was required to activate the mPT (118). Here, 20 μM PAO and 400 μM CaCl_2_ (1 mM MgCl_2_, pH 7.3, 37 °C, 45 min) increased OG-HTL labeling to ~94% of singlets (Fig. 4*I*), which we interpreted as the maximal response.

After OG-HT staining and mPT activation, we fixed cells to increase throughput. Since permeabilization reduces cell morphology information (SSC-A vs. FSC-A), fixation methods were optimized with intact cells and OGAc_2_-HTL on a flow cytometer. With each day after fixation, cell size (FSC-A) drifted smaller and gated particle counts were reduced (FSC-A, *SI Appendix*, Fig. S7*D*). Peak OGAc_2_-HTL fluorescence shifted darker over time; however, gated particle counts were unchanged (*SI Appendix*, Fig. S7*E*). Thus, fixation did not damage the fluorophore. Results were similar with permeabilization, mPT activation, and a cell sorter. Cells were fluorescently stable 24 h after fixation and persisted for 5 d (Fig. 4*J*).

### Genome-Wide CRISPR-Cas9 FACS Screen for the Mitochondrial Permeability Assay.

**Fig. 5.**
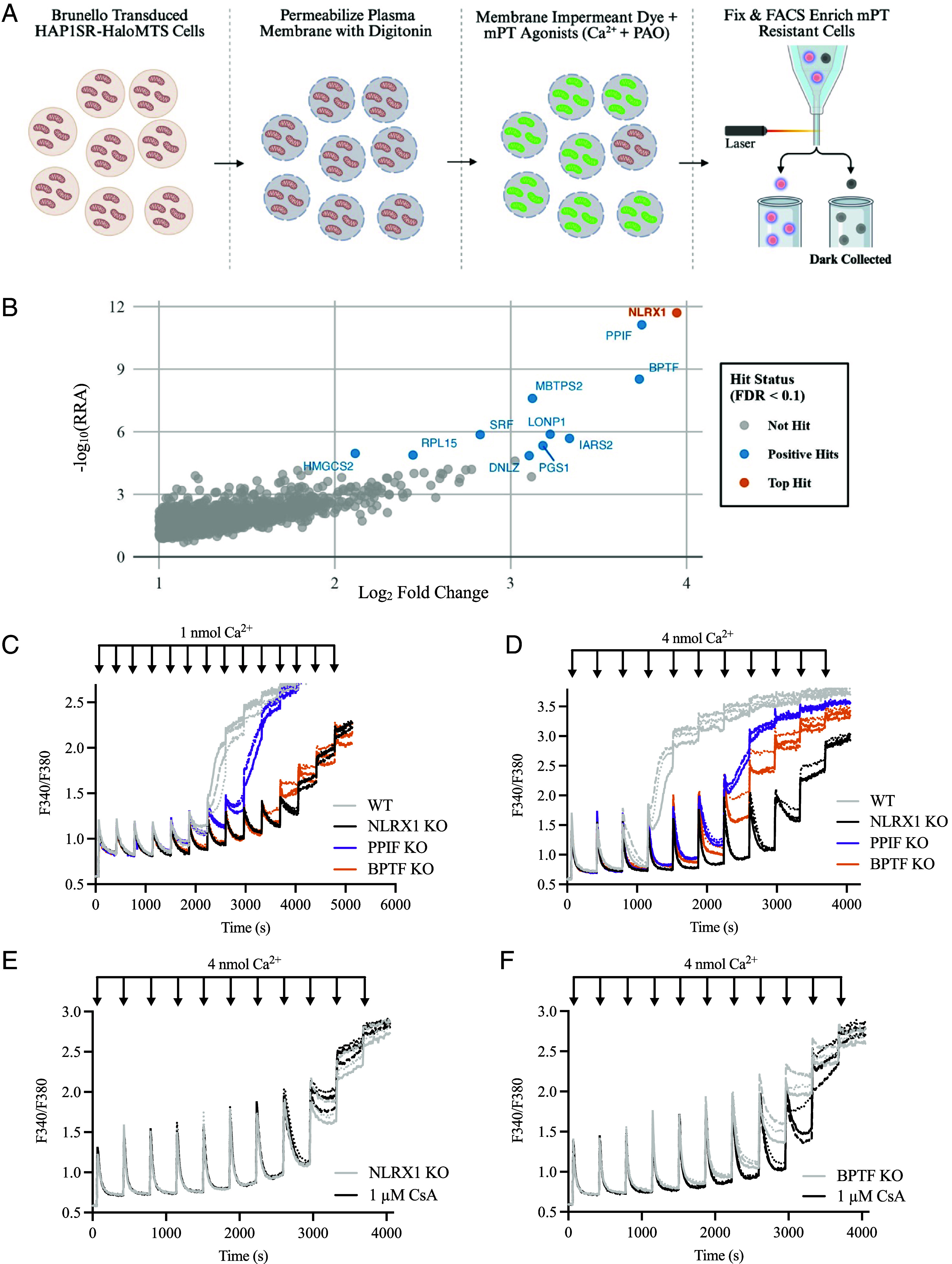
The top hit of the genome-wide CRISPR-Cas9 FACS screen for the mitochondrial permeability assay, NLRX1, is an mPT activator. (*A*) Schematic of the genome-wide CRISPR-Cas9. HAP1SR-HaloMTS cells were transduced with the Brunello library at low MOI. Cellular plasma membranes were digitonin-permeabilized in the standard mitochondrial solution, washed, then incubated with impermeant (synthesized) Oregon Green HaloTag ligand (30 nM) and mPT agonists (400 μM Ca^2+^ and 20 μM PAO) for 45 min (37 °C, 0% CO_2_). Next, cells were washed, fixed (4% PFA, 30 min, ice) washed, and then sorted by FACS (beginning 24 h after fixation). Cells resistant to the mPT agonists (dark cells) were collected for positive sgRNA enrichment analysis. (*B*) NLRX1 (orange) is the top statistical hit. Screen analysis was performed using MAGeCK RRA and plotted as -log_10_(RRA) vs. Log_2_ Fold Change (LFC). The volcano plot is cropped at LFC > 1.0 to show positive hits (FDR < 0.1; blue and orange). (C and D) NLRX1 KO and BPTF KO increase CRC and prevent Ca^2+^ release. Ca^2+^ boluses of 1 nmol (*C*) or 4 nmol (*D*) were added to WT (gray), NLRX1 KO (black), PPIF KO (purple), and BPTF KO (orange) until Ca^2+^ uptake failed or Ca^2+^ release occurred. (*E* and *F*) Cyclosporin A (CsA) is ineffective in NLRX1 KO. CRC comparing control (gray) and 1 μM CsA (black) in NLRX1 KO (*E*) and BPTF KO (*F*) with 4 nmol Ca^2+^ boluses until Ca^2+^ uptake failed or Ca^2+^ release occurred. In (*C*–*F*), digitonin-permeabilized HAP1 cells were seeded at 2 million cells/well in a 96-well blackout plate for Ca^2+^ imaging (1 μM Fura-FF; F340/F380).

The mitochondrial permeability assay was applied to Brunello expressing HAP1SR-HaloMTS cells (Fig. 5*A*). Beginning 24 h after fixation, ~235 million events were recorded by a cell sorter and ~4.5 million dark particles were collected across 4 d. NGS data were analyzed by the MAGeCK RRA package (109) and passed quality control metrics. Zero sgRNA counts were 255 (99.7% coverage), the Gini Index was 0.069, and mapped reads/sgRNA were over 3,900 in control samples (day of experiment).

In total, 19,113 genes were assessed from 77,186 sgRNAs. With a statistical threshold of FDR < 0.1 (110) and LFC > 1.0, 11 genetic hits were identified, ranked by positive RRA score (Dataset S2), and plotted by -log_10_(RRA) x LFC (Fig. 5*B*). The dataset revealed three exceptional hits: mitochondrial matrix NLRX1, mPT sensitizer PPIF (70–72), and nuclear chromatin binding BPTF (125). Surprisingly, only one (nonmitochondrial MBTPS2) of the 11 hits was an unequivocal integral membrane protein. We also did not expect EMRE or MCU to be hits because the deleterious rise of matrix Ca^2+^ (and associated mPT) does not require rapid Ca^2+^ entry (50, 126). GSEA across Protein Complexes, GO Biological Process, KEGG, and Reactome (111) did not reveal any significantly enriched positive pathways (*SI Appendix*, Fig. S8). Compared to the prior screen, LFC enrichment was greater in top-ranked hits. The failure of GSEA to identify established pathways suggests new pathways exist or the results comprise essential single genes.

### NLRX1 Is a Critically Important Activator of the mPT.

We studied the positively enriched RRA hits by single gene KO (HAP1 cells) with CRC and UCR. Several mitochondrial hits, including the LONP1 protease (5th), IARS2 (mitochondrial tRNA ligase, 7th), PGS1 (cardiolipin synthesis, 8th), and HSPA9-binding DNLZ (11th) could not be genetically disrupted and expanded as clonal cell lines, suggesting that these genes are required for HAP1 cell propagation. Genetic disruption of nonmitochondrial MBTPS2 (Zn^2+^ metalloprotease, 4th) and mitochondrial HMGCS2 (ketogenic enzyme, 9th, *SI Appendix*, Fig. S9*A*) did not alter CRC (*SI Appendix*, Fig. S9 *B* and *C*) or UCR (*SI Appendix*, Fig. S9 *D* and *E*), respectively. Lower ranked nonmitochondrial hits (6th, 9th, 10th) were not evaluated.

NLRX1 KO increased CRC more than BPTF KO or PPIF KO (Fig. 5*C*). In NLRX1 KO and BPTF KO, Ca^2+^ release did not occur, and CRC proceeded until Ca^2+^ uptake failed (Fig. 5*C*). During Ca^2+^ uptake failure and Ca^2+^ signal stacking, the Ca^2+^ signal showed a small upward drift in both genotypes. However, this was often observed in HAP1 cells after the mPT, including the parallel recorded WT and PPIF KO (Fig. 5*C*), and was attributed to a non-mPT process. To increase confidence, CRC experiments were repeated with Ca^2+^ boluses that were 4-fold larger. The CRC increase was still the largest in NRLX1 KO, still observed with BPTF KO, and Ca^2+^ release did not occur (Fig. 5*D*).

CsA-sensitivity in the CRC or swelling assays persisted in the prior studies that challenged the proposed mPT candidates under KO (85–91). Here, 1 μM CsA expectedly increased the CRC of WT HAP1 cells (*SI Appendix*, Fig. S10*A*), did not increase the CRC of PPIF KO HAP1 cells (*SI Appendix*, Fig. S10*B*), and Ca^2+^ release was unaltered in both genotypes (*SI Appendix*, Fig. S10 *A* and *B*). In NLRX1 KO, 1 μM CsA did not increase CRC or stimulate a Ca^2+^ release event, and the CRC traces were mostly superimposed (Fig. 5*E*). In BPTF KO, 1 μM CsA increased CRC and likely stimulated a Ca^2+^ release event (Fig. 5*F*). We conclude that NRLX1 is an essential activator of the CsA-sensitive human mPT and BPTF is a potentiator or sensitizer.

Each candidate was evaluated in the UCR assay. In response to 5 μM CCCP, Ca^2+^ release was delayed in KOs, but never prevented (*SI Appendix*, Fig. S10*C*). The magnitude of the delay was the largest in NLRX1 and PPIF KOs, aligning with their rankings in the genome-wide screen. Since NLRX1 KO was effective at preventing the CsA-sensitive mPT, but ineffective at preventing UCR, the UCR may be independent of the mPT. Alternatively, CCCP may be a stronger Ca^2+^ release stimulus than Ca^2+^ uptake, and thus, Ca^2+^ release is only delayed in NLRX1 KO. In this latter interpretation, the mPT and UCR share a biological mechanism.

## Discussion

The mPT is a mitochondrial Ca^2+^ overload response that permeabilizes the IMM (36–39). Several mitochondrial integral membrane proteins have been proposed as genetic candidates for the mPT (76–84), but all have been disputed (85–91). The purpose of this study was to uncover the essential proteins in mitochondrial Ca^2+^ overload and the human mPT in an unbiased manner. By applying phenotypic genome-wide CRISPR-Cas9 screening to high-throughput assays, four protein-encoding genes were identified and validated: NF2, REST, BPTF, and NRLX1. Genetic disruption of BPTF, NF2, or REST increased CRC, but either did not prevent Ca^2+^ release or were CsA-sensitive. These nonmitochondrial genes fit the classification of mPT sensitizers. Mitochondrial NLRX1, the top-ranked hit of the mitochondrial permeability screen, was the only KO to abolish Ca^2+^ release and exhibit no CsA-sensitivity, meeting the definition of an essential mPT activator (85–91).

The MMP screen strongly enriched the essential proteins of rapid mitochondria Ca^2+^ influx. No KO prevented large-scale Ca^2+^ release via the mPT, and most did not alter CRC. The connection between MMP collapse and the mPT has been challenging to study in mammalian mitochondria because MMP collapse occurs alongside Ca^2+^ release (105, 106). In mammalian mitochondria, it has also been proposed that membrane depolarizations activate or are required for the mPT (127, 128). However, in *Drosophila melanogaster*, MMP collapsed during Ca^2+^ uptake failure even when the mPT did not activate (118). Our results in human mitochondria align with the *Drosophila melanogaster* observation. We suggest that ionomycin shuttled Ca^2+^ into the cytoplasm and the mitochondrial electrochemical gradient facilitated large-scale rapid Ca^2+^ entry. When SMDT1 or MCU were genetically disrupted, rapid Ca^2+^ influx was blunted, and the MMP collapse was prevented or delayed beyond our collection period. Therefore, MMP collapse is not a reporter of the mPT.

The purposes and consequences of the mPT have been difficult to assess without critical proteins identified. Early reports suggested NLRX1, the only Nod-Like Receptor (NLR) targeted to mitochondria (129), had an OMM-based immune function through MAVS (130). However, other studies detected NRLX1 in the matrix (131, 132), not the OMM (133), and MAVS-dependent antiviral signaling was not depressed in NLRX1 KO (134). Functionally, mitochondrial-localized NLRX1 overexpression increased ROS production (135). In SV40 transformed mouse embryonic fibroblasts, NLRX1 KO was correlated with decreased ROS and protection against Ca^2+^ ionophore (A23187) mediated apoptosis (136). Depressed ROS production and Ca^2+^ ionophore apoptotic resistance align with a desensitized or abrogated mPT.

There are several pathological NLRX1 KO studies on immune dysfunction and inflammation (137–140). However, the mPT is strongly associated with IR injury (45–52, 57, 58). Genetic disruption of NLRX1 worsened IR injury (141–143), notably for “severe” IR injury (35 min ischemia, Langendorff perfusion) (142). In humans, NRLX1 protein expression was decreased in patients with acute myocardial ischemia (144). During our hit validation, NLRX1 KO and the mouse mPT were studied in cardiac mitochondria with IR injury (141). NLRX1 KO increased CRC, prevented Ca^2+^ release, and the CRC was CsA-insensitive. Moreover, basal mitochondrial [Ca^2+^] was unchanged in NLRX1 KO, yet was substantially increased post-IR, further suggesting the mPT had not activated (141). Whether ROS levels differ in NRLX1 KO IR injury is less clear. ROS levels were higher in a renal IR model (143) and unchanged in a cardiac IR model (141); however, the methodologies used were inconsistent. Nonetheless, mouse studies conclude that NLRX1 KO exacerbates IR injury (141–143), potentially by preventing mPT activation (141).

In addition to mammals (37–39, 85, 106) and *Drosophila melanogaster* (118, 119), the mPT has been detected in *Saccharomyces cerevisiae* (145) and the plant, pea stem (*Pisum sativum*) (146). The mPT has been reported in *Caenorhabditis elegans* (147), but CRC or swelling assays have not been performed. *Saccharomyces cerevisiae* is a challenging organism to study the mPT due to the absence of Ca^2+^ uptake mechanisms (148, 149) and the MCU gene (14). The proposed mPT, measured by absorbance-based swelling after ionophore-induced Ca^2+^ uptake, was permeable to PEG molecules up to 2,000 Da (145), similar to mammalian mitochondria (37, 91, 150). However, differing pharmacological properties were noted, including the absence of CsA sensitivity (145, 151). The mPT of *Saccharomyces cerevisiae* implies that Ca^2+^ is not the primary mPT activator. Instead, Ca^2+^ may initiate a cascade that increases production of a direct mPT activator, such as ROS (152).

Crustaceans, including *Crangon crangon*, *Palaemon serratus,* and the embryos of *Artemia franciscana,* may not undergo a mPT (153, 154). These organisms did not swell or release Ca^2+^ at saturating levels of Ca^2+^ uptake (153, 154). In both studies, Ca^2+^ ionophores or PAO were not used, and thus it is uncertain if the mPT is not present or if it is desensitized beyond the limit of Ca^2+^ uptake, like *Drosophila melanogaster* (118). Coincidentally, adult *Drosophila melanogaster* (155) and embryonic *Artemia franciscana* (156) decrease metabolic rates during anoxic periods lasting hours (155) or years (157) by ~97% (158) and ~99.97% (156), respectively. This confers substantial resistance to IR (ROS) injury. Without injury, there may be no evolutionary benefit to NLRX1, which invertebrates do not express; NLRX1 evolved in jawed vertebrates (159). We hypothesize that the ubiquitously expressed (135) NLRX1 evolved alongside organisms that are vulnerable to short-duration anoxia and NLRX1 mitigates tissue damage by activating the mPT.

Using patch clamp electrophysiology with protein purification and reconstitution, two integral IMM proteins were proposed as proteinaceous mPTP candidates. The first, bovine ANT, elicited 300 to 600 pS single channel events that recapitulated some mPT pharmacology (77). The second, gel-purified F_0_F_1_ ATP synthase dimers, elicited maximal conductances of ~1,000 pS (81). Human ATP5G1, a subunit of the F_0_F_1_ ATP synthase c-ring, generated multiconductance recordings of 100 pS to ~2,000 pS (82). Independent KO studies with phenotypic assays did not corroborate these candidate proposals (85, 86, 89–91), yet these proteins are still described as putative mPTP candidates in the literature.

The incongruent patch clamp and phenotypic data are expected when patch clamp controls are inadequate. Mitoplast patch clamp data for the proposed mPTP presented conductances ranging from 10 pS to 2,700 pS (160–162). Associations between the large conductances and the mPT relied on cofactors and pharmacology (163–165). They were also inconsistent, as one study reported Ca^2+^-inhibited currents (166). Technical issues were stated, including seal collapse at voltages above ±50 mV (161), which is not a mitoplast patch clamp limitation (12, 112, 167–169). Seal instability (170) was present in recordings, including high frequency and oscillatory noise during open events (161–163, 166). This body of work renders reconstitution studies susceptible to false positives, as unequivocal identification of protein(s) requires a defined single channel conductance, current modification by protein pore mutagenesis (171), and/or specific pore blockers with defined site-specific actions. None of these exist for the mPTP.

A recent preprint proposed ATAD3 as a “putative pore-forming component” of the mPTP in mouse heart and liver mitochondria (172). ATAD3 KO abolished CsA-sensitive mPT phenotypic outputs at the limit of endogenous Ca^2+^ uptake (172). Mouse ATAD3 (ATAD3A) is conserved in mammals (173), however, humans have two additional paralogs (174), ATAD3B (175, 176) and ATAD3C (177). ATAD3B and ATAD3A reportedly hetero-oligomerize (178), are codetected in mass spectrometry pulldowns (179), and are ubiquitously expressed (180). Consequently, the ATAD3 paralogs may be functional heterooligomers. In our screens, all ATAD3 paralogs were detected, but none were hits. We hypothesize that ATAD3A KO will not prevent the human mPT. If the mouse ATAD3 KO mPT effects translate to humans, triple ATAD3 KO may be required.

Our study has limitations. First, all single-gene CRISPR KO screens are limited by gene redundancy or KO compensation. If the hypothetical proteinaceous mPTP arises from redundant entities, they may not be detected in our study. KO compensation may mask sensitizers. For example, KO compensation has been reported for the ANT paralogs (86), likely explaining why these established sensitizers were not enriched in the mitochondrial permeability screen. Second, genomes contain small open reading frames (sORFs) that transcribe microproteins (181), which were not evaluated in our study. Recent annotations estimate 2500-7500 sORFs in the human genome (182, 183). These sORFs could be regulators of mitochondrial Ca^2+^ overload, like Mitoregulin (184). Third, the mitochondrial permeability assay utilized an exogenously expressed mitochondrial HaloTag protein and enriched candidates were sorted by the absence of fluorescence. Positively enriched hits could arise from protection against the mPT or from failed expression of the mitochondrial HaloTag protein. This could explain why LONP1, IARS2, PGS1, and DNLZ were enriched in the permeability screen, yet each was detrimental to clonal propagation under KO.

Notwithstanding these limitations, our work demonstrates the essential requirement of NLRX1 to the human mPT and questions if integral membrane proteins underlie the process. We propose three possibilities for the mPT. First, NLRX1 could be the “transmembrane hydrophilic channel” (37) of the mPT. NLRX1 is a matrix protein with tight association to the IMM (132) and potentially hexameric in structure (185, 186). However, there is no evidence of membrane insertion for NLRX1. Second, NRLX1 may be a scaffold protein (187) that recruits a redundant pore-forming complex to the IMM, similar to the NLRP3 inflammasome and Gasdermin D (188–190). However, the pore-forming protein should be in the mitochondrial matrix, and none have been identified.

The third, and our preferred, hypothesis is that NLRX1 is a ROS potentiator creating peroxidized lipid pores. NLRX1 immunoprecipitation mass spectrometry identified UQCRC2, a matrix facing Complex III protein, as a potential interactor (132). In mouse hepatocytes, NLRX1 KO increased electron transport chain (ETC) O_2_ consumption rate (191). NLRX1 knockdown also increased Complex I and III redox activities (192) and NLRX1 KO decreased ROS (136). We speculate that NLRX1 alters ETC (redox center) conformations, slows forward electron transport and/or induces reverse electron transport (152). This increases electron leakage and ROS, and the localized spike in ROS generates discrete peroxidized lipid pores. In support, molecular dynamic simulations show peroxidized lipids can form ~1.5 nm diameter hydrophilic pores (193, 194), similar to the mPT pore estimates (91, 195). Highly curved membranes are also more vulnerable to lipid peroxidation (196). Ca^2+^ may function as an agonist for ROS, as elevated matrix Ca^2+^ increased CsA-sensitive mtROS before mPT activation (152). ROS peroxidized lipid pores would also resolve discrepancies in the mPT literature since they would offer an explanation for the Ca^2+^ desensitized mPT in anoxia-resistant (and NLRX1 nonexpressing) invertebrates (118, 119, 153, 154), the mPT in native Ca^2+^-uptake incapable *Saccharomyces cerevisiae* (145, 148, 149, 151), and the absence of top-ranked IMM proteins in our mitochondrial permeability screen.

## Materials and Methods

HAP1 cells were cultured according to manufacturer recommendations. Individual gene KO were generated by transient transfection of ribonucleoprotein complexes (CRISPR-Cas9). Genome-wide KO were generated by lentiviral transduction of the pooled Brunello library (108). HaloMTS (mitochondrial HaloTag protein) was stably expressed by lentiviral transduction. Flow cytometry and FACS-enriched populations were first gated for cell morphology, then singlets, then the fluorescent reporter. Confocal microscopy was performed on a Zeiss LSM 880 microscope using a plan apochromat 63x/1.4 NA oil DIC M27 objective lens.

The MMP FACS assay utilized a customized rhodamine voltage dye (JF_549_M) and mitochondrial Ca^2+^ overload was induced by the Ca^2+^ ionophore, ionomycin. The mitochondrial permeability FACS assay was receptor–ligand format, using a mitochondrial localized HaloTag protein and a membrane impermeant Oregon Green HaloTag ligand dye in permeabilized cells. The mPT was activated by Ca^2+^ and PAO. The mPT and mitochondrial Ca^2+^ overload were independently measured with CRC and UCR assays in permeabilized cells (89–91, 105, 106). Extramitochondrial Ca^2+^ was empirically titrated and fluorescence measured by Fura-FF in a 96-well plate reader.

For full details of these methods, see *SI Appendix*.

## Supplementary Material

Appendix 01 (PDF)

Dataset S01 (XLSX)

Dataset S02 (XLSX)

## Data Availability

FASTQ files data have been deposited in European Nucleotide Archive (ENA) (PRJEB101006) (197). Study data are included in the article and/or supporting information.
