# Supplementary material for: Phenotypic CRISPR screens identify NLRX1 as an essential activator of the human mitochondrial permeability transition
Source: Proc Natl Acad Sci U S A. 2026 Feb 25;123(9):e2535298123. doi: 10.1073/pnas.2535298123 (PMC12956895; doi:10.1073/pnas.2535298123)
Supplement: Supplementary file 1 — Appendix 01 (PDF) [file pnas.2535298123.sapp.pdf]

SI Appendix for:

Phenotypic CRISPR screens identify NLRX1 as an essential activator of the human mitochondrial permeability transition

William C. Valinsky[1], Robert P. Ray[1], Kathy S. Schaefer[1], Jonathan B. Grimm[1], Carla Nicolini[1], Luke D. Lavis[1], and David E. Clapham[1,2]

[1]Janelia Research Campus, Howard Hughes Medical Institute, Ashburn, VA 20147, USA

[2]To whom correspondence may be addressed. Email: claphamd@hhmi.org

Materials and Methods

Cell Culture. HAP1 (Horizon Discovery C631) and HAP1 Screening Ready (HAP1SR; Horizon Discovery C859) cells were grown in Iscove's Modified Dulbecco's Medium (ATCC 30-2005) supplemented with 10% Fetal Bovine Serum (FBS) Tetracycline Negative (Gemini Bio 100-800) in a CO_2 incubator (Thermo Fisher Scientific, Heracell VIOS 160i) at 37°C in 5% CO_2. Cells were sub-cultured every 2-4 days in a sterile biosafety cabinet with confluency maintained below 75%, per Horizon Discovery instructions. In cell engineering experiments, low passage cells were utilized. Cells were propagated for at least one-week post-thaw before experimentation.

The following HAP1 KO cell lines were obtained from Horizon Discovery:
FBXW7 KO (HZGHC003212c008)
NF2 KO (HZGHC001293c001)
ANO6 KO (HZGHC002956c003)
GLO1 KO (HZGHC006372c008)
REST KO (HZGHC006341c008)
ESYT1 KO (HZGHC003669c011)
MICU1 KO (HZGHC006829c008)
NLRX1 KO (HZGH79671)
PPIF KO (HZGHC004185c012)
BPTF KO (HZGHC001369c010)
MBTPS2 KO (HZGHC001523c010)

Lenti-X-293T cells (Takara 632180) were grown in Dulbecco's Modified Eagle's Medium (DMEM) with high glucose (4.5 g/L), L-glutamine, and $NaHCO_3$ (Sigma-Aldrich D5796) supplemented with 10% FBS Tetracycline Negative Cells (Gemini Bio 100-800) and 1 mM Na-pyruvate (Sigma-Aldrich S8636) in a CO_2 incubator (Thermo Fisher Scientific, Heracell VIOS 160i) at 37°C in 5% CO_2. Cells were sub-cultured every 2-4 days in a sterile biosafety cabinet.

Cells were frequently tested for mycoplasma (Invivogen rep-mys-50 or ATCC 136-XV). In the event of positive result, cells were discarded or recovered with Plasmocin (Invivogen mpt-apt).

Flow Cytometry and FACS. Genetically modified cell lines, either CRISPR-Cas9 non-homologous end joining (NHEJ) frameshift indels or stable ectopic protein expression, were created in this study. For CRISPR-Cas9 NHEJ frameshift indels, positively transfected cells were selected and seeded by FACS. All enrichments were performed using a cell sorter (Sony Biotechnology SH800S, 100 μm chip). Events were first gated for cell morphology (backscatter

– area; BSC-A vs. forward scatter – area, FSC-A), then gated for single cells (singlets; FSC-A vs. forward scatter – width, FSC-W), then gated for the fluorescent reporter in the green (FSC-A vs. fluorescein isothiocyanate – area, FITC-A; 488 nm laser) or red (FSC-A vs. phycoerythrin – area, PE-A; 561 nm laser) channels. Non-transfected control cells were used to establish 'dark' fluorescent gates. A 'bright' gate was established to identify positively transfected cells, at minimum ¼ log brighter than dark. Photomultiplier tube voltages and sorting pressure were optimized in protocol development and then rarely changed. Bright singlets were sorted into 96-well plates in fresh media using single-cell mode (one cell per well). The 96th well contained 50-100 cells to monitor sorting fidelity. For stable protein expression, an identical gating strategy was used, however, cells were collected in bulk with a fluorescent cut-off (top 5% brightness) in ultra-purity sorting mode.

Assays to study mitochondrial Ca^{2+} overload and the mPT were designed to be compliant with FACS. These assays were initially optimized on a flow cytometer in 96-well plate mode acquisition (Beckman Coulter CytoFLEX). Events were first gated for cell morphology (side scatter – area, SSC-A vs. FSC-A), then gated for single cells (singlets; FSC-A vs. FSC-W), then gated for the fluorescent reporter in the green (FSC-A vs. FITC-A; 488 laser) or red (FSC-A vs. PE-A; 561 nm laser) channels. Final optimizations were performed on a cell sorter (Sony Biotechnology SH800S, 100 μm chip) to design gates, optimize voltages, and evaluate sorting modes.

Figure panels were produced in FlowJo (v10.10.0). Figure gates were reproduced to match populations as they were depicted on the cytometer or cell sorter.

Transient and Stable Cell Line Generation. For transient expression, cDNA was transfected in 35 mm dishes using Lipofectamine 2000 (Thermo Fisher Scientific 11668027) according to manufacturer's guidelines. Briefly, 2000 ng of cDNA was mixed with 4 μL of Lipofectamine 2000 in 250 μL of Opti-MEM (Thermo Fisher Scientific 31985062). The DNA-lipid complexes were added to cells in 2 mL of fresh media and incubated for ~48 h before use.

For the genome-wide permeability screen, HAP1SR cells stably expressed a mitochondrial HaloTag protein. The mitochondrial HaloTag insert of Halo-MTS (Addgene 124315) was subcloned into a lentiviral transfer plasmid with a UCOE-SFFV (1) promoter using Gibson Assembly (NEB E2621). Lentivirus was produced in Lenti-X-293T cells (Takara 632180) by co-transfection (Lipofectamine 2000, Thermo Fisher Scientific, 11668027) of the lentiviral transfer plasmid with psPAX2 (Addgene 12260) and pMD2.G (Addgene 12259) in a 4:3:1 molar ratio, 24 h after cell seeding (60% confluency). Approximately 72 h after transfection, the supernatant was collected, centrifuged (1000 rpm, 5 min, 4°C), and low passage HAP1SR cells were

transduced in fresh IMDM + FBS media containing polybrene (8 µg/mL, EMD Millipore TR-1003-G). To enrich a population of high-expressing cells, HAP1SR-HaloMTS cells were treated with membrane permeant 30 nM acetylated Oregon Green HaloTag ($OGAc_2$-HTL; Promega G2801) and the brightest 5% of cells were enriched by FACS (FSC-A vs. FITC-A; 488 nm laser). After recovery, cells were sorted a second time with the brightest 5% collected for further enrichment.

Chemical Synthesis. Commercial reagents were obtained from reputable suppliers and used as received. All solvents were purchased in septum-sealed bottles stored under an inert atmosphere. All reactions were sealed with septa through which a nitrogen atmosphere was introduced unless otherwise noted. Reactions were conducted in round-bottomed flasks or septum-capped crimp-top vials containing Teflon-coated magnetic stir bars. Heating of reactions was accomplished with a silicon oil bath or an aluminum reaction block on top of a stirring hotplate equipped with an electronic contact thermometer to maintain the indicated temperatures.

Reactions were monitored by thin layer chromatography (TLC) on precoated TLC glass plates (silica gel 60 F_{254}, 250 µm thickness) or by LC/MS (Phenomenex Kinetex 2.1 mm × 30 mm 2.6 µm C18 column; 5 µL injection; 5–98% MeCN/H_2O, linear gradient, with constant 0.1% v/v HCO_2H additive; 6 min run; 0.5 mL/min flow; ESI; positive ion mode). TLC chromatograms were visualized by UV illumination or developed with *p*-anisaldehyde, ceric ammonium molybdate, or $KMnO_4$ stain. Reaction products were purified by flash chromatography on an automated purification system using pre-packed silica gel columns or by preparative HPLC (Phenomenex Gemini–NX 30 × 150 mm 5 µm C18 column). Analytical HPLC analysis was performed with an Agilent Eclipse XDB 4.6 × 150 mm 5 µm C18 column under the indicated conditions. High-resolution mass spectrometry was performed by the High-Resolution Mass Spectrometry Facility at the University of Iowa and HHMI Janelia Mass Spectrometry.

NMR spectra were recorded on a 400 MHz spectrometer. ^{1}H and ^{13}C chemical shifts were referenced to TMS or residual solvent peaks, and ^{19}F chemical shifts were referenced to $CFCl_3$. Data for ^{1}H NMR spectra are reported as follows: chemical shift (δ ppm), multiplicity (s = singlet, d = doublet, t = triplet, q = quartet, dd = doublet of doublets, m = multiplet), coupling constant (Hz), integration. Data for ^{13}C NMR spectra are reported by chemical shift (δ ppm) with hydrogen multiplicity (C, CH, CH_2, CH_3) information obtained from DEPT spectra.

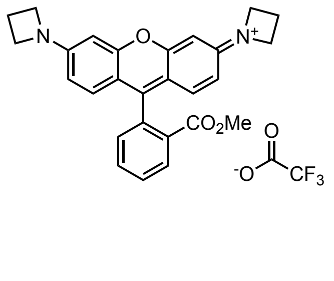

3,6-Di(azetidin-1-yl)-9-(2-(methoxycarbonyl)phenyl)xanthylium trifluoroacetate ($JF_{549}M$): To a solution of JF_{549} (50 mg, 0.122 mmol) in CH_2Cl_2 (5 mL) was added oxalyl chloride (41.2 µL, 0.487 mmol, 4 eq). After stirring the reaction at room temperature for 30 min, MeOH (1 mL) and Et_3N (136 µL, 0.974 mmol, 8 eq) were added in succession. The reaction was stirred at room temperature for 1 h, concentrated *in vacuo*, and purified by reverse phase HPLC (30–60% $MeCN/H_2O$, linear gradient, with constant 0.1% v/v TFA additive) to yield 64.7 mg (99%) of $JF_{549}M$ (**7**) as a dark red solid. ^1H NMR (CD_3OD, 400 MHz) δ 8.33 – 8.28 (m, 1H), 7.86 (td, J = 7.5, 1.4 Hz, 1H), 7.80 (td, J = 7.7, 1.4 Hz, 1H), 7.43 – 7.38 (m, 1H), 7.04 (d, J = 9.2 Hz, 2H), 6.59 (dd, J = 9.2, 2.2 Hz, 2H), 6.53 (d, J = 2.2 Hz, 2H), 4.33 – 4.27 (m, 8H), 3.61 (s, 3H), 2.56 (p, J = 7.7 Hz, 4H); ^{13}C NMR (CD_3OD, 101 MHz) δ 166.9 (C), 160.7 (C), 158.8 (C), 158.1 (C), 135.3 (C), 134.1 (CH), 132.24 (CH), 132.19 (CH), 131.59 (CH), 131.49 (CH), 131.41 (C), 114.9 (C), 113.6 (CH), 95.1 (CH), 52.9 (CH_3), 52.8 (CH_2), 16.8 (CH_2); Analytical HPLC: t_R = 11.8 min, >99% purity (10–95% $MeCN/H_2O$, linear gradient, with constant 0.1% v/v TFA additive; 20 min run; 1 mL/min flow; ESI; positive ion mode; detection at 555 nm); HRMS (ESI) calculated for $C_{27}H_{25}N_2O_3$ $[M]^+$ 425.1860, found 425.1863.

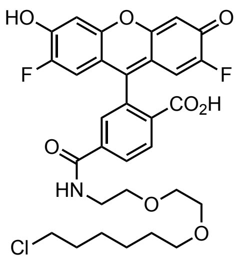

Oregon Green HaloTag ligand (OG–HTL): Oregon Green 488 carboxylic acid succinimidyl ester, 6-isomer (OG-NHS; 20 mg, 39.3 µmol) and 2-(2-((6-chlorohexyl)oxy)ethoxy)ethanamine hydrochloride (HaloTag(O2)amine; 20.4 mg, 78.5 µmol, 2 eq) were combined in DMF (3 mL), and DIEA (34.2 µL, 0.196 mmol, 5 eq) was added. After stirring the reaction at room temperature for 1 h, it was concentrated *in vacuo* and purified by reverse phase HPLC (10–95% $MeCN/H_2O$, linear gradient, with constant 0.1% v/v TFA additive) to afford 22.9 mg (94%) of Oregon Green HaloTag ligand (**13**) as a yellow-orange solid. ^1H NMR (CD_3OD, 400 MHz) δ 8.17 (dd, J = 8.0, 1.4 Hz, 1H), 8.13 (d, J = 8.1 Hz, 2H), 7.68 – 7.64 (m, 1H), 6.86 (d, $^4J_{HF}$ = 7.4 Hz, 2H), 6.47 (d, $^3J_{HF}$ = 11.1 Hz, 2H), 3.63 – 3.55 (m, 4H), 3.55 – 3.48 (m, 6H), 3.39 (t, J = 6.5 Hz, 2H), 1.75 – 1.66 (m, 2H), 1.52 – 1.43 (m, 2H), 1.43 – 1.35 (m, 2H), 1.34 – 1.27 (m, 2H); ^{19}F NMR (CD_3OD, 376 MHz) δ -139.12 – -142.41 (m); Analytical HPLC: t_R = 13.1 min, >99% purity (10–95% $MeCN/H_2O$, linear gradient, with constant 0.1% v/v TFA additive; 20 min run; 1 mL/min flow; ESI; positive ion mode; detection at 280 nm); HRMS (ESI) calculated for $C_{31}H_{31}ClF_2NO_8$ $[M+H]^+$ 618.1701, found 618.1701.

Confocal Microscopy. Cells were seeded onto glass bottom dishes (Cellvis, #D35-20-1.5-N) 24-48 h prior to imaging. Dyes were applied to cells in fresh media to ensure accurate concentrations (15-30 min, 37°C, 5% CO_2). After incubation, cell media was aspirated and cells were washed twice with phenol-free Hanks' Balance Salt Solution with or without Ca^{2+} and Mg^{2+}, where appropriate. Prior to the synthesis of a membrane impermeant Oregon Green HaloTag (OG-HTL), we generated an impermeant variant of $OGAc_2$-HTL (Promega G2801) by cell media incubation at RT for at least 3 h (2).

The following dyes were used: MitoTracker Green (100 nM; Thermo Fisher Scientific M7514), Rhod-2, AM, cell permeant (5 μM; Thermo Fisher Scientific R1245MP), Hoechst 33342 (1 μg/mL; Thermo Fisher Scientific H3570), Tetramethylrhodamine, Methyl Ester, Perchlorate (TMRM, 25 nM, Thermo Fisher Scientific T668), $JF_{549}M$ (25 nM), $OGAc_2$-HTL (30 nM; Promega G2801), OG-HTL (30 nM), JF_{549}-HTL (100 nM). When reconstitution was necessary, desiccated dyes were brought to RT and solubilized with fresh, high quality dimethyl sulfoxide (Sigma-Aldrich, D2650).

Images were captured on a Zeiss LSM 880 microscope using a plan apochromat 63x/1.4 NA oil DIC M27 objective lens. Cells were placed in an imaging chamber at 37°C with atmospheric CO_2 and located with brightfield. Fluorescence was confirmed by epifluorescence under low intensity. For laser scanning confocal images, excitation was 405 nm for Hoechst 33342, 488 nm for MitoTracker Green and Oregon Green, and 561 nm for Rhod-2, TMRM, JF_{549}-HTL, and $JF_{549}M$. Laser power was kept under 2% to limit photodamage and gain of 600-800 arbitrary units was adjusted to maximize signal while preventing histogram saturation. Images of 1584 × 1584 pixels were collected at 16-bit, scanned at 4-line averages, with pinhole size (Air Units) automatically adjusted by Zeiss Zen.

For time-lapse recordings, stage drift was minimized using the hardware-based focus stabilization feature, Definite Focus. Images were captured every 30-60 s to ensure adequate image acquisition time and minimize photodamage.

Analysis was performed in Fiji (Image J, v1.54p). For sample images, min/max was adjusted manually to brighten images. Scale bars were also added. For tracking fluorescence over time, an empirically determined threshold was applied to images to selectively capture mitochondria. Next, a binary mask was applied to the threshold image, and all particles were analyzed based on empirically determined size and circularity parameters. Finally, fluorescence over time was analyzed across the time series using the multi-measure function.

Genome-Wide CRISPR Screening. For genome-wide screening, the Brunello library (3) was utilized (Addgene, one plasmid lentiCRISPRV2 73179-LV; titer ~1.5E7 TU/mL in both screens). Protocols from Doench (3) and Addgene were followed to generate a cell library with one lentiviral particle per cell and suitable coverage/depth. Briefly, a puromycin kill curve was generated for each cell line such that ~99% of cells died by day 3 (HAP1) or day 4 (HAP1SR-HaloMTS), with antibiotic replenishment every 48 h. Puromycin (Gemini Bio 400-128P) doses were 2 μg/mL and 1 μg/mL, respectively.

To obtain the multiplicity of infection (MOI), 3 million cells were seeded in each well of a 12-well plate with 8 μg/mL polybrene (EMD Millipore TR-1003-G). Cells were transduced with lentiCRISPRV2 at 0 μL, 50 μL, 100 μL, 200 μL, and 250 μL in duplicate (10 wells), spinfected at 1000 g for 2 h at 33°C, and incubated at 37°C, 5% CO_2 for ~24 h. Next, cells were expanded to 15 cm dishes, grown for 24 h, and one of the duplicates (5 plates) was selected with the optimized puromycin dose. Transduction efficiency for a given viral volume was calculated as the percentage of living transduced cells with puromycin to living transduced cells without puromycin. The viral amount corresponding to a transduction efficiency of 30-50%, a MOI of ~0.35-0.70, was chosen for genome-wide experimentation. Death of non-transduced cells (0 μL lentivirus) was used to assess the fidelity of the antibiotic.

Library transductions were performed with low passage cells to mitigate spontaneous HAP1 diploid transformations (4). Transduced cell counts were estimated to produce at minimum 400 cells/sgRNA (5) post-selection. Based on the transduction efficiency calculation, ~95-160 million input cells were transduced using the methods described above. Post-selection, 45 million surviving cells (T_0) were frozen for analysis of initial coverage (~580 cells/sgRNA). At each passage, cells were pooled and seeded to maintain coverage of at least 400 cells/sgRNA. On experimentation days, 45 million cells (T_3 for MMP, T_2 for mitochondrial permeability) were frozen as coverage/count controls. Experiments were performed within 4 passages of Brunello library generation to minimize sgRNA dropout (6).

Mitochondrial Membrane Potential FACS Assay. In the genome-wide screening experiment, cells were split, centrifuged (1000 rpm, 5 min, RT) and resuspended in Hanks' Balanced Salt Solution with Ca^{2+}, Mg^{2+}, and phenol red free (HBSS Ca^{2+}/Mg^{2+}; Gibco 14025092) + 20 mM HEPES (pH 7.3 with NaOH) at 10^6 cells/mL. $JF_{549}M$ (25 nM) was added, mixed, and cells incubated for 15 min (37°C, 0% CO_2). Next, 10 μM ionomycin (Millipore Sigma 407950) was added, mixed, and returned to the incubator. After the 5 min incubation, cells were centrifuged (1000 rpm, 5 min, 4°C) and resuspended (10 million/mL) in fresh HBSS Ca^{2+}/Mg^{2+} + 20 mM HEPES (pH 7.3 with NaOH) on ice.

The assay was optimized using flow cytometry. MMP was empirically gated for dark and bright particles using WT HAP1 cells (FSC-A vs. PE-A; 561 nm laser). Gates for living cells (SSC-A vs. FSC-A), singlets (FSC-A vs. FSC-W), and MMP were modestly adjusted in each experiment; voltages and gate shape rarely changed. In control conditions, cell morphology comprised ~85% of all events and singlets comprised ~95-99% of cell morphology counts. $JF_{549}M$ did not alter cell morphology counts or singlets. [Ionomycin] was evaluated with 5 μM, 7 μM, 10 μM, 15 μM, 20 μM, 30 μM, or 40 μM; 10 μM ionomycin showed maximum $JF_{549}M$ quenching without extensive cell death.

In a mock cell sorting experiment where the sample was held at 4°C, 10 μM ionomycin depleted $JF_{549}M$ fluorescence (~92% to 1%), cell morphology slightly declined (~85% to ~80%), and singlets were unchanged. These parameters were mostly stable for 60 min. By 90 min, gated living cell counts and $JF_{549}M$ fluorescence were in decline. The empirically determined sorting limit before abort counts rose was ~2000 events/s (7.2 million events/h). To maintain coverage of ~400 cells/sgRNA, ~31 million input cells were required. To comply with the viability time limit (60 min), the Brunello-HAP1 cells were processed in batches of 5-7 million. To minimize variance between processing, two cell sorters (Sony Biotechnology SH800S, 100 μm chip) were concurrently utilized. With 3 batches of cells, 6 FACS experiments were performed.

In the genome-wide screen (Brunello-HAP1 cells), experimental fidelity was confirmed using parallel processed WT HAP1-$JF_{549}M$ cells with control and ionomycin-treated conditions. Ionomycin-treated singlets were semi-yield sorted for the top 1% $JF_{549}M$ fluorescence (FSC-A vs. PE-A; 561 nm laser). Throughout the experiment, the $JF_{549}M$ bright gate was adjusted to maintain top 1% brightness collection. The sample and collection were held at 4°C. Sorted contents were collected in an Eppendorf at 4°C with an ~100 μL bed volume of PBS, and after each round of collection, were centrifuged (13000 g, 5 min, 4°C) with pellets snap frozen in liquid nitrogen. The schematic for the assay was built in BioRender with a publication license.

Mitochondrial Permeability FACS Assay. HAP1SR-HaloMTS cells were cultured for 48 h, split, and resuspended (10 million/mL) in a standard mitochondrial solution with the plasma membrane permeabilizing agent, digitonin. The standard mitochondrial solution contained 125 mM KCl, 2 mM K_2HPO_4, 1 mM $MgCl_2$, 10 μM EGTA, 20 mM HEPES, 5 mM L-malate, and 5 mM L-glutamate (pH 7.3 with KOH, ~300 mOsm with sucrose). For plasma membrane permeabilization (7), 50 ng/μL digitonin (Promega G9441) and 0.5 mM EDTA were added. Cells were incubated for at least 5 min on ice. Permeabilization was monitored by cell counting with Trypan blue stain (Invitrogen T10282) in a cell counter (Countess II, Thermo Fisher Scientific) and deemed complete when >99% of cells were Trypan blue positive. Permeabilized cells were

centrifuged (200 g, 10 min, 4°C) and resuspended (10 million/mL) in the standard mitochondrial solution with 30 nM OG-HTL on ice.

The assay was optimized using flow cytometry. Permeabilization resulted in one large population by cell morphology (SSC-A or BSC-A vs. FSC-A) and singlets (FSC-A vs. FSC-W). In addition, permeabilized cell morphology measurements were smaller than intact cells. In preliminary experiments with $OGAc_2$-HTL, fluorescence was detected throughout the cell morphology population. Thus, gates were designed to capture as many particles as possible, with only the smallest particles (presumed fragments) excluded. The fluorescent channel (OG-HTL; FSC-A vs. FITC-A; 488 nm laser) was empirically gated for dark and bright singlets using control WT HAP1SR-HaloMTS cells. Where stated in this section, control refers to parallel processing and incubation; only the mPT agonists (400 μM $CaCl_2$ and 20 μM PAO) were excluded.

The following parameters were tested to maximize mPT activation (OG-HTL fluorescence) in isolation or combination: $CaCl_2$ (400 μM, 2 mM), $MgCl_2$ (0 mM, 1 mM), pH (7.3, 7.8, 8.3, 8.8), tert-butyl hydroperoxide (7 μM, 70 μM, 700 μM, 7 mM, 70 mM), PAO (20 μM), incubation time (45 min, 90 min), and incubation temperature (RT, 37°C). Many variables did not meaningfully change fluorescence but high dose tert-butyl hydroperoxide eliminated the fluorescent signal. The combination of variables that elicited consistent OG-HTL fluorescence of ~95% in mPT activation conditions (compared to ~1% control) were: 400 μM $CaCl_2$, 20 μM PAO, 1 mM $MgCl_2$, pH 7.3, 45 min incubation, 37°C.

After the above incubation, cells were centrifuged (500 g, 10 min, 4°C), washed (ice cold standard mitochondrial solution containing no dye or mPT agonists), and centrifuged again (500 g, 10 min, 4°C). Next, cells were resuspended and fixed with fresh 4% PFA (0.1 M phosphate buffer) at 1 million cells/mL for 30 min on ice, centrifuged (2000 g, 10 min, 4°C), washed (ice cold PBS + 0.5% BSA), and centrifuged again (2000 g, 10 min, 4°C). Cells were resuspended to 15 million/mL in PBS + 0.5% BSA and stored in a dark environment at 4°C until use.

FACS enrichment (Sony Biotechnology SH800S, 100 μm chip) was performed in normal purity mode. The sample and collection were held at 4°C. Gates (morphology, singlets, OG-HTL fluorescence) were same-day optimized using parallel processed WT HAP1SR-HaloMTS cells (control and mPT agonist treated), which also confirmed methodological fidelity in the genome-wide experiment. In the sort, mPT agonist-treated Brunello HAP1SR-HaloMTS cells were collected from the OG-HTL dark gate because they were protected from mPT activation. Sorted contents were collected in an Eppendorf tube with a bed volume of 100 μL PBS + 0.5% BSA, centrifuged (2500 g, 30 min, 4°C), and frozen at -80°C. The schematic for the assay was built in BioRender with a publication license.

Genomic DNA Processing, Next-Generation Sequencing (NGS), and Analysis. Genomic DNA was extracted with Machery-Nagel NucleoSpin blood kits without protocol modification. Column size was selected such that cell counts were near the midpoint of column capacity. For fixed samples, an initial de-crosslinking step was performed prior to gDNA isolation with 1 mg/mL Proteinase K for ~6 h.

PCR reactions (Clontech RR001A; Takara Biosciences) with up to 10 μg genomic DNA per reaction were performed to amplify sgRNAs and attach the necessary sequencing adaptors for Illumina next-generation sequencing (NGS). Primers (P5 & P7) were synthesized by IDT using guidance from Addgene and the Broad Institute: https://media.addgene.org/cms/filer_public/61/16/611619f4-0926-4a07-b5c7-e286a8ecf7f5/broadgpp-sequencing-protocol.pdf.

PCR cycling conditions were optimized to balance yield with purity: 95°C for 1 min, 95°C for 30 s (denaturation), 63°C for 30 s (annealing), and 72°C for 30 s (extension) for a total of 22 cycles. A final extension at 72°C lasted 10 min. Samples were pooled after PCR. Non-specific background was evaluated by Bioanalyzer (Agilent), with target product of ~285 bp.

SPRI beads, using the double SPRI protocol with SPRIselect (Beckman Coulter B23317), were utilized to remove contaminants of the PCR reaction prior to NGS (Illumina NextSeq 2000). Briefly, DNA above 300 bp was bound to beads at 0.65X, washed, and supernatant transferred to a new microcentrifuge tube containing 1X SPRI beads (binds >150 bp products), washed, and eluted with 20 μL of elution buffer. Product purity was evaluated by Bioanalyzer (Agilent). Purified PCR products were diluted to 750 pM for NGS and loaded with a 5% PhiX spike-in. NextSeq 2000 flow cells had read capacities of 100 million (MMP assay) or 1.2 billion (mitochondrial permeability assay).

NGS data was analyzed using MAGeCK Robust Rank Algorithm (RRA) (8) in Python, with hits ranked based on positive RRA scores. Screen quality was assessed by coverage (zero sgRNA count), Gini Coefficient (target below 0.1 in controls), depth (minimum 80 reads per sgRNA), and mapped read percentage (target >60%). Figure panels were produced in GraphPad Prism except for the mitochondrial permeability screen volcano plot, which was produced in R (v4.5.1) using ggplot2 and ggrepel packages. Hits were classified by false discovery rate (FDR) < 0.1, with secondary confirmation by $\log_2$-fold change (LFC) > 1.0. Data were filtered using dplyr to visually maximize positive hits and eliminate all data where LFC < 1.0.

MAGeCKFlute (v.1.99.0) (9) in R (v4.5.1) used the RRA datasets to perform gene set enrichment analysis (GSEA) across KEGG, GOBP, Reactome, and Complex (CORUM). Figures for GSEA were produced in R (v4.5.1) with ggplot2 and patchwork. The top 5 pathways per

category were plotted and ranked by adjusted p-value ($-\log_{10}$FDR), with statistical significance presented as a dashed orange line at $-\log_{10}$FDR = 1.3 (FDR < 0.05).

Single Gene CRISPR-Cas9 Non-Homologous End Joining. Where possible, HAP1 KOs were purchased from Horizon Discovery. Otherwise, KOs were made using the IDT Alt-R platform. With IDT Alt-R, sgRNAs were generated by annealing synthesized CRISPR RNA (crRNA) to transactivating CRISPR RNA (tracrRNA ATTO 488; IDT 10007810 or tracrRNA ATTO 550; IDT 1075928) in equimolar amounts (95°C, 5 min; 1 μM final). Next, ribonucleoprotein (RNP) complexes were produced by combining equimolar purified *Streptococcus pyogenes* Cas9 Nuclease V3 (IDT 1081058) with sgRNAs for 5 min, RT. Last, RNP complexes (10 nM final) were transfected into 400,000 HAP1 cells using RNAiMAX (Thermo Fisher Scientific, 13778075) in each well of a 96-well plate (150 μL total volume). The following day cells were split, and successfully transfected cells were enriched by FACS using fluorescence from ATTO488 or ATTO550. Non-transfected WT cells were parallel sorted to act as controls for fluorescent gating, sorting fidelity, and downstream CRC/UCR controls.

Genomic DNA from ~50,000 cells was extracted by QuickExtract (Lucigen QE905T) following manufacturer protocols. sgRNA containing amplicons were PCR amplified (Platinum SuperFi II, Thermo Fisher Scientific, 12368010) using customized primer sets (IDT) and empirically evaluated annealing temperatures. Crude PCR products were purified (NEB Monarch T1030L) and confirmed pure by agarose gel electrophoresis prior to Sanger sequencing. Candidate KOs were evaluated by Sanger sequencing chromatograms in Benchling (aligned with the *H. sapiens* genes) or DECODR (10). KOs were selected by frameshift mutations (indels) near the expected cut-site.

In our FACS-based workflow, single transfected cells were seeded into at least two 96-well plates, per gene. For genes where KO failed, all surviving clones were WT or heterozygotes (minimum 190 wells). When this occurred, colony recovery was far lower than control. As heterozygotes (multiple alleles) should be rare in low passage HAP1 cells (haploid cells), we hypothesized that homozygous disruption of these genes interfered with cell propagation.

crRNAs:

SMDT1: GGATCACAATGGAGAACACA
MCU: GGAGCTTATTGAAAGACTAG
DFFA: GCCACAAACTTAGTATTGGA
EWSR1: TGGGTCTTCATAGGACACTG
FAM193A: GGTCCATGATACCACACGTG

RPRD1B: GTGGATGAGCCAAAGGGACA
TRPM4: GCCGAGTAGTTGTAGTCCAG
HMGCS2: ATCCACTATTGGGTACTCCG

Calcium Retention Capacity (CRC) and Uncoupling Response (UCR) Assays. The CRC assay is a bulk measurement assay that monitors Ca^{2+} uptake, overload, and release with an extramitochondrial Ca^{2+} dye of low μM K_D (11-22). Dual simultaneous measurements in mammalian mitochondria demonstrated a correlation between Ca^{2+} release and absorbance-based swelling (19, 21, 22) or MMP collapse (15, 20), thus integrating multiple mPT phenotypes. In circumstances where Ca^{2+} release is prevented in mammalian mitochondria, CRC pulse counts increase, but Ca^{2+} uptake eventually fails (19). At failure, the Ca^{2+} signal stacks, rather than forming a large continuous jump, and absorbance-based swelling does not occur (19). The large Ca^{2+} release event is employed as the mPT reporter in the CRC assay.

Cells were prepared in a similar manner to the Mitochondrial Permeability FACS Assay. Cells were split and resuspended in a digitonin (50 ng/μL) containing standard mitochondrial solution (ice cold) at 10 million cells/mL, with permeabilization monitored by Trypan blue staining. Next, cells were centrifuged and resuspended at 20 million cells/mL in an ice cold standard mitochondrial solution containing 1 μM Fura-FF (Cayman Chemical Company 20415).

For CRC experiments, permeabilized cells were seeded in 96-well blackout plates (Corning 3603) in 100 μL increments (2 million cells per well) and acclimatized to RT over 5 min. Plates were loaded into a Spark Microplate Reader with injectors (Tecan Group Ltd.). Permeabilized cells were subjected to a Ca^{2+} cycling protocol. In brief, Fura-FF fluorescence (A.U.) was recorded for 6 min (Ca^{2+}-bound: 340 nm excitation, 507 nm emission; Ca^{2+}-free: 380 nm excitation, 514 nm emission), followed by injection of the standard mitochondrial solution containing $CaCl_2$. This was repeated until Ca^{2+} release events were detected or Ca^{2+} uptake failed. Where indicated, cyclosporin A (CsA; Sigma Aldrich 30024) was added to RT samples for a 5 min incubation period immediately prior to recording.

For the uncoupler-induced Ca^{2+} release (UCR) assay, cell preparation was identical to above. The UCR protocol consisted of a single, larger injected bolus of the standard mitochondrial solution containing $CaCl_2$, with 1 μM Fura-FF monitored for 6 min. A mitochondrial uncoupler containing standard mitochondrial solution (carbonyl cyanide m-chlorophenylhydrazone, CCCP; 5 μM final; Sigma C2759) was injected, followed by a 5-15 min recording.

In candidate CRC/UCR experiments, a paired WT control was utilized in each experiment. We noted that the absolute CRC in WT cells was dependent on various culture conditions including days of culture post-thaw, post-split, and seeding density. Thus, paired controls and seed counted

cells minimized confounding variables. To ensure accuracy within a well, all experiments were recorded in technical triplicates. The minimum sampling rate (per well) for any recording was 6 events per 60 s.

Fura-FF signal normalization (F340/F380) was performed in Microsoft Excel and all subsequent figures created using GraphPad Prism. Normalized CRC (KO/Control) was determined by counting the number of Ca^{2+} pulses until a release event occurred. Each triplicate from a single experiment was averaged, generating a single CRC quantification. The CRC counts for each KO were divided by the paired control, resulting in a fraction. KOs where meaningful increases in normalized CRC were observed (>~1.25) were repeated, resulting in a minimum of 3 independent datasets (mean ± S.D.).

References

1. F. Zhang *et al.*, A ubiquitous chromatin opening element (UCOE) confers resistance to DNA methylation-mediated silencing of lentiviral vectors. *Mol Ther* **18**, 1640-1649 (2010).
2. L. D. Lavis, T. Y. Chao, R. T. Raines, Synthesis and utility of fluorogenic acetoxymethyl ethers. *Chem Sci* **2**, 521-530 (2011).
3. J. G. Doench *et al.*, Optimized sgRNA design to maximize activity and minimize off-target effects of CRISPR-Cas9. *Nat Biotechnol* **34**, 184-191 (2016).
4. T. B. Beigl, I. Kjosas, E. Seljeseth, N. Glomnes, H. Aksnes, Efficient and crucial quality control of HAP1 cell ploidy status. *Biol Open* **9** (2020).
5. O. Shalem *et al.*, Genome-scale CRISPR-Cas9 knockout screening in human cells. *Science* **343**, 84-87 (2014).
6. B. C. Cross *et al.*, Increasing the performance of pooled CRISPR-Cas9 drop-out screening. *Sci Rep* **6**, 31782 (2016).
7. I. Schulz, Permeabilizing cells: some methods and applications for the study of intracellular processes. *Methods Enzymol* **192**, 280-300 (1990).
8. W. Li *et al.*, MAGeCK enables robust identification of essential genes from genome-scale CRISPR/Cas9 knockout screens. *Genome Biol* **15**, 554 (2014).
9. B. Wang *et al.*, Integrative analysis of pooled CRISPR genetic screens using MAGeCKFlute. *Nat Protoc* **14**, 756-780 (2019).
10. K. Bloh *et al.*, Deconvolution of Complex DNA Repair (DECODR): Establishing a Novel Deconvolution Algorithm for Comprehensive Analysis of CRISPR-Edited Sanger Sequencing Data. *CRISPR J* **4**, 120-131 (2021).
11. J. E. Kokoszka *et al.*, The ADP/ATP translocator is not essential for the mitochondrial permeability transition pore. *Nature* **427**, 461-465 (2004).
12. C. P. Baines *et al.*, Loss of cyclophilin D reveals a critical role for mitochondrial permeability transition in cell death. *Nature* **434**, 658-662 (2005).
13. T. Nakagawa *et al.*, Cyclophilin D-dependent mitochondrial permeability transition regulates some necrotic but not apoptotic cell death. *Nature* **434**, 652-658 (2005).

14. M. Gutierrez-Aguilar *et al.*, Genetic manipulation of the cardiac mitochondrial phosphate carrier does not affect permeability transition. *J Mol Cell Cardiol* **72**, 316-325 (2014).
15. S. Shanmughapriya *et al.*, SPG7 Is an Essential and Conserved Component of the Mitochondrial Permeability Transition Pore. *Mol Cell* **60**, 47-62 (2015).
16. J. He, J. Carroll, S. Ding, I. M. Fearnley, J. E. Walker, Permeability transition in human mitochondria persists in the absence of peripheral stalk subunits of ATP synthase. *Proc Natl Acad Sci U S A* **114**, 9086-9091 (2017).
17. J. He *et al.*, Persistence of the mitochondrial permeability transition in the absence of subunit c of human ATP synthase. *Proc Natl Acad Sci U S A* **114**, 3409-3414 (2017).
18. J. Carroll, J. He, S. Ding, I. M. Fearnley, J. E. Walker, Persistence of the permeability transition pore in human mitochondria devoid of an assembled ATP synthase. *Proc Natl Acad Sci U S A* **116**, 12816-12821 (2019).
19. J. Karch *et al.*, Inhibition of mitochondrial permeability transition by deletion of the ANT family and CypD. *Sci Adv* **5**, eaaw4597 (2019).
20. K. Mallilankaraman *et al.*, MCUR1 is an essential component of mitochondrial Ca2+ uptake that regulates cellular metabolism. *Nat Cell Biol* **14**, 1336-1343 (2012).
21. M. J. Bround *et al.*, ANT-dependent MPTP underlies necrotic myofiber death in muscular dystrophy. *Sci Adv* **9**, eadi2767 (2023).
22. P. Patel *et al.*, The adenine nucleotide translocase family underlies cardiac ischemia-reperfusion injury through the mitochondrial permeability pore independently of cyclophilin D. *Sci Adv* **10**, eadp7444 (2024).

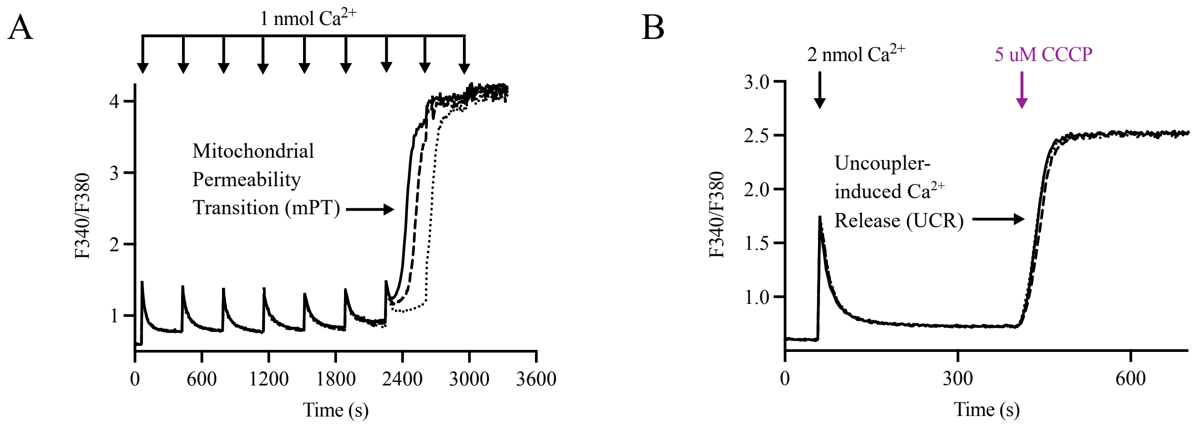

Fig. S1. Large-scale Ca^{2+} release in HAP1 mitochondria. Representative Ca^{2+} retention capacity (CRC; A) and uncoupler-induced Ca^{2+} release (UCR; B) traces in technical triplicates (n = 3; solid line, dashed line, dotted line). In CRC (A), 1 nmol Ca^{2+} boluses were added until the Ca^{2+} release event (the mPT) was complete. In UCR (B), a single 2 nmol Ca^{2+} bolus was added, followed by 5 μM uncoupler (CCCP), and fluorescence recorded until Ca^{2+} release (the UCR) was complete. Digitonin-permeabilized HAP1 cells were seeded at 2 million cells/well in a 96-well blackout plate for Ca^{2+} imaging (1 μM Fura-FF; F340/F380).

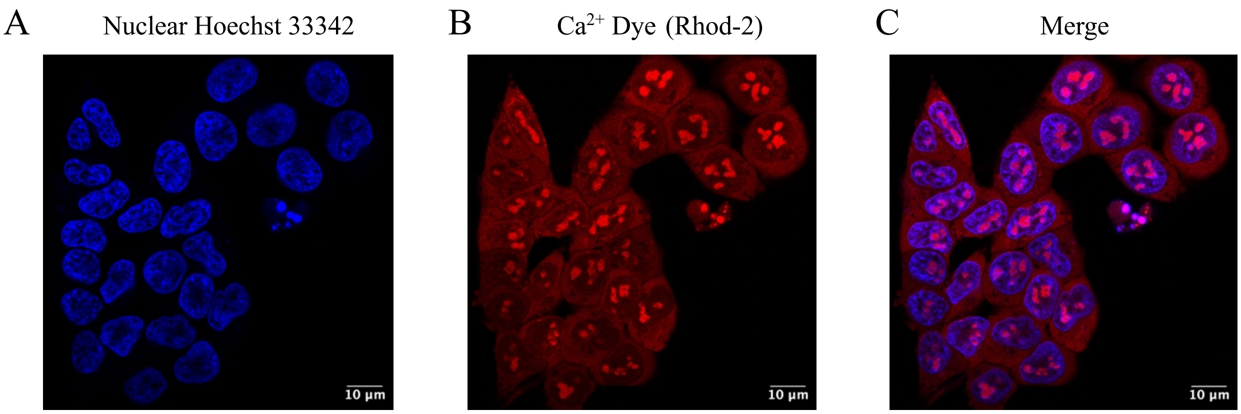

Fig. S2. HAP1 cells have a Rhod-2 nuclear signal. Confocal imaging (63X) of intact WT HAP1 cells treated with the nuclear stain Hoechst 33342 (1 µg/mL, blue) and the Ca^{2+} indicator Rhod-2, AM (5 µM, red). Split channel images are shown for Hoechst 33342 (A) and Rhod-2 (B); merged in (C). Scale bars = 10 µm.

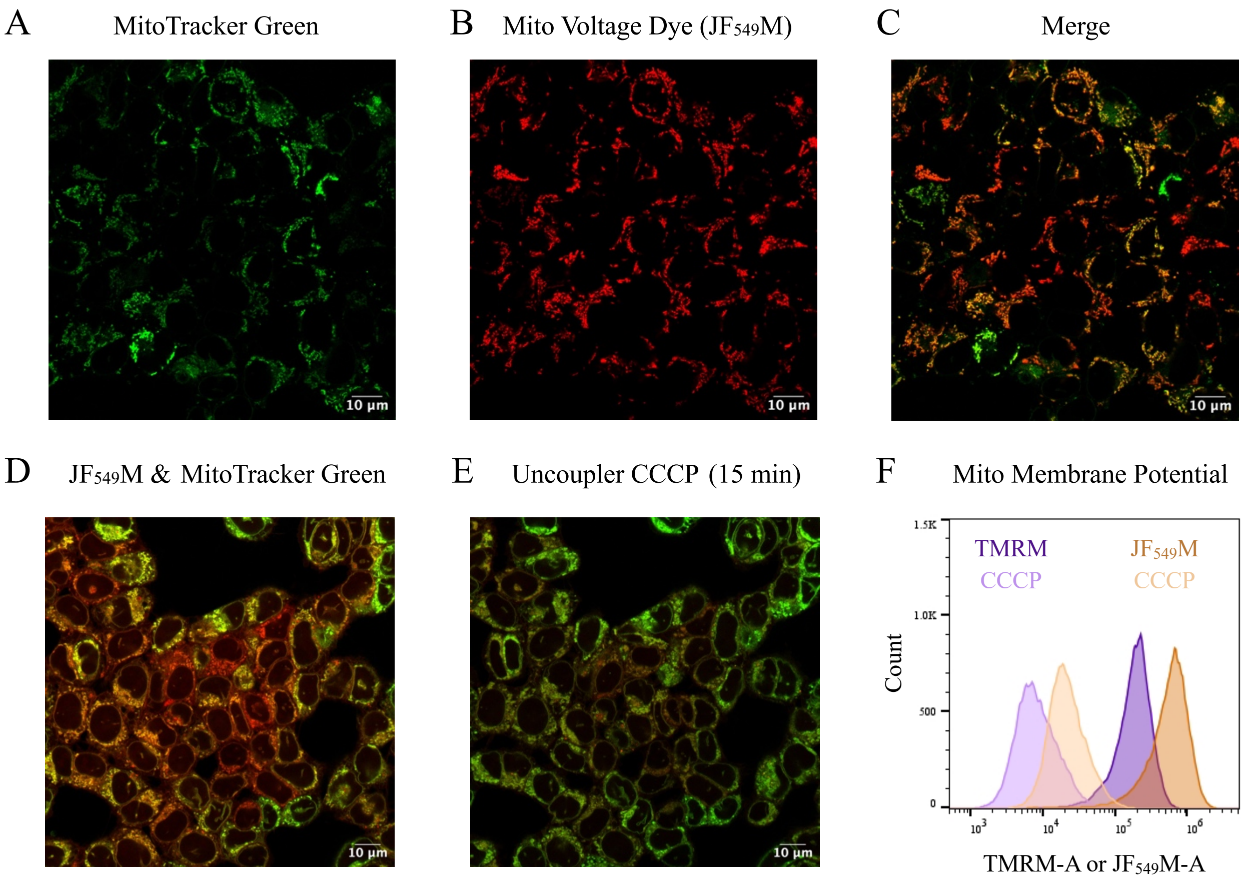

Fig. S3. $JF_{549}M$ is a mitochondrial voltage dye and brighter than TMRM. (A-C) Confocal imaging (63X) of intact HAP1 cells treated with 100 nM MitoTracker Green and the rhodamine-based mitochondrial voltage dye, $JF_{549}M$ (25 nM). Split channel images are shown for MitoTracker Green (A) and $JF_{549}M$ (B); merged in (C). Scale bars = 10 µm. The protonophore and mitochondrial uncoupler CCCP depletes $JF_{549}M$ fluorescence (D and E). Confocal images (63X) of intact WT HAP1 cells treated with 100 nM MitoTracker Green and 25 nM $JF_{549}M$ before (D) and 15 min after 5 µM CCCP application (E). Images were recorded in a time series experiment. Scale bars = 10 µm. $JF_{549}M$ is brighter than TMRM (F). Flow cytometry histograms comparing 25 nM $JF_{549}M$ (orange) and 25 nM TMRM (purple) in control (dark, right) and 5 µM CCCP (light, left) conditions. Particles were first gated for living cells (SSC-A vs. FSC-A), then singlets (FSC-A vs. FSC-W), then fluorophore brightness. TMRM-A and $JF_{549}M$-A shared laser excitation of 561 nm.

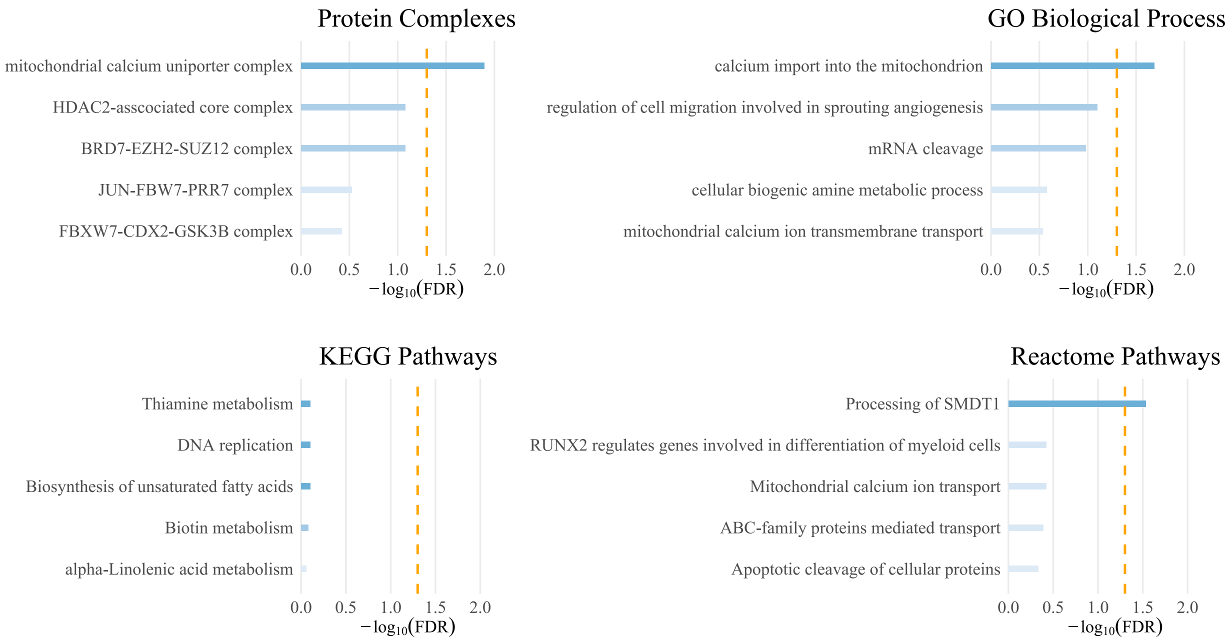

Fig. S4. Gene set enrichment analysis (GSEA) for the mitochondrial membrane potential CRISPR screen. Pathways related to the mitochondrial Ca^{2+} uniporter complex are enriched. GSEA was performed on genes ranked by positive RRA, and enrichment was tested across Protein Complexes, GO Biological Processes, KEGG, and Reactome Pathways. The top five pathways plotted by -$\log_{10}$(FDR). Statistical significance (-$\log_{10}$(FDR) = 1.3; FDR < 0.05) is denoted by the dashed orange line.

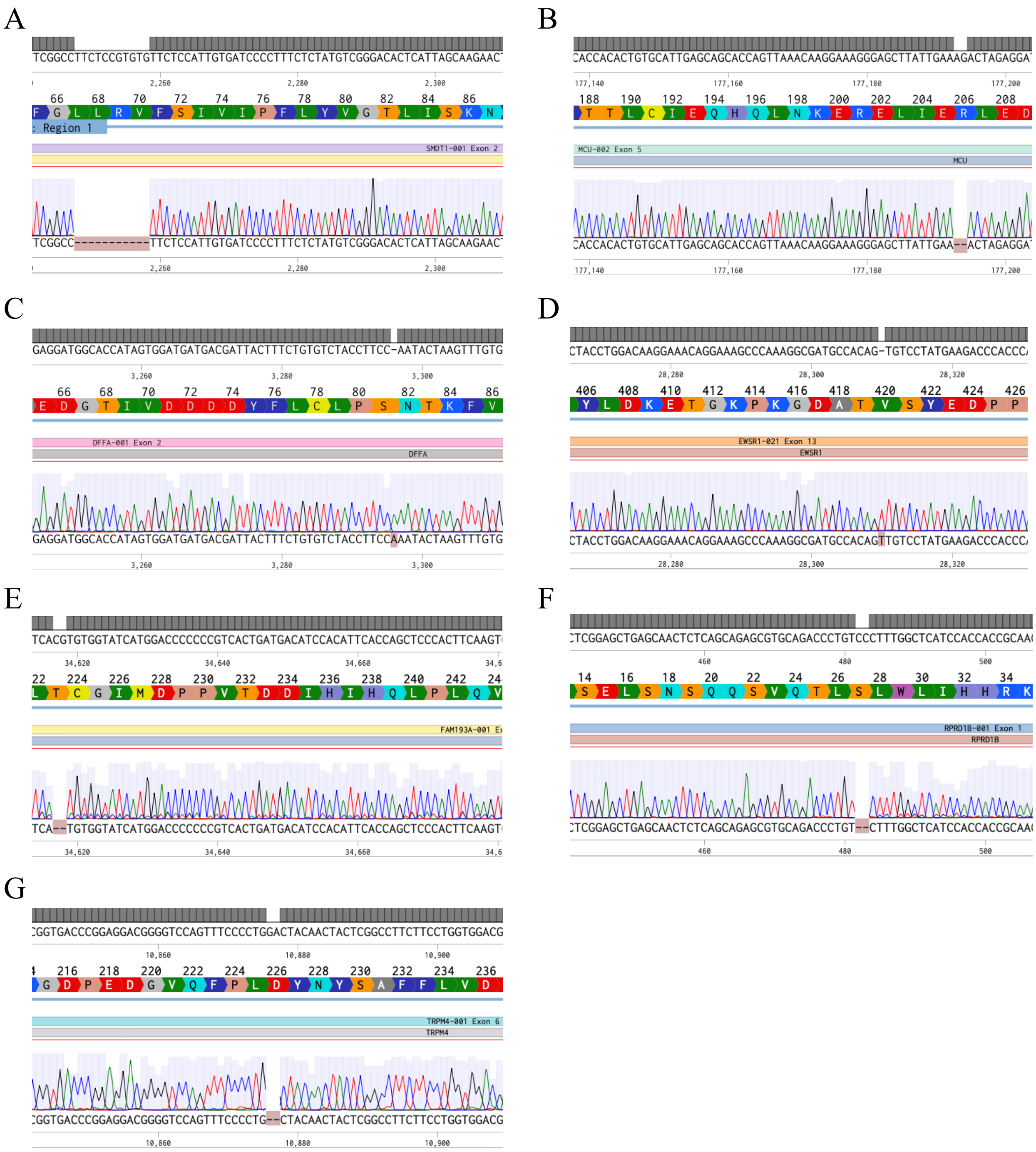

Fig. S5. Sanger sequencing verification of clonal knockouts. Using ~50,000 cells from each monoclonal population, gDNA was isolated, regions around the sgRNA amplicons were PCR amplified, purified by column filtration, and sequenced by Sanger sequencing to identify frameshift indels. Chromatograms for SMDT1 (A), MCU (B), DFFA (C), EWSR1 (D), FAM193A (E), RPRD1B (F), and TRPM4 (G) were aligned with *Homo sapiens* genes in Benchling.

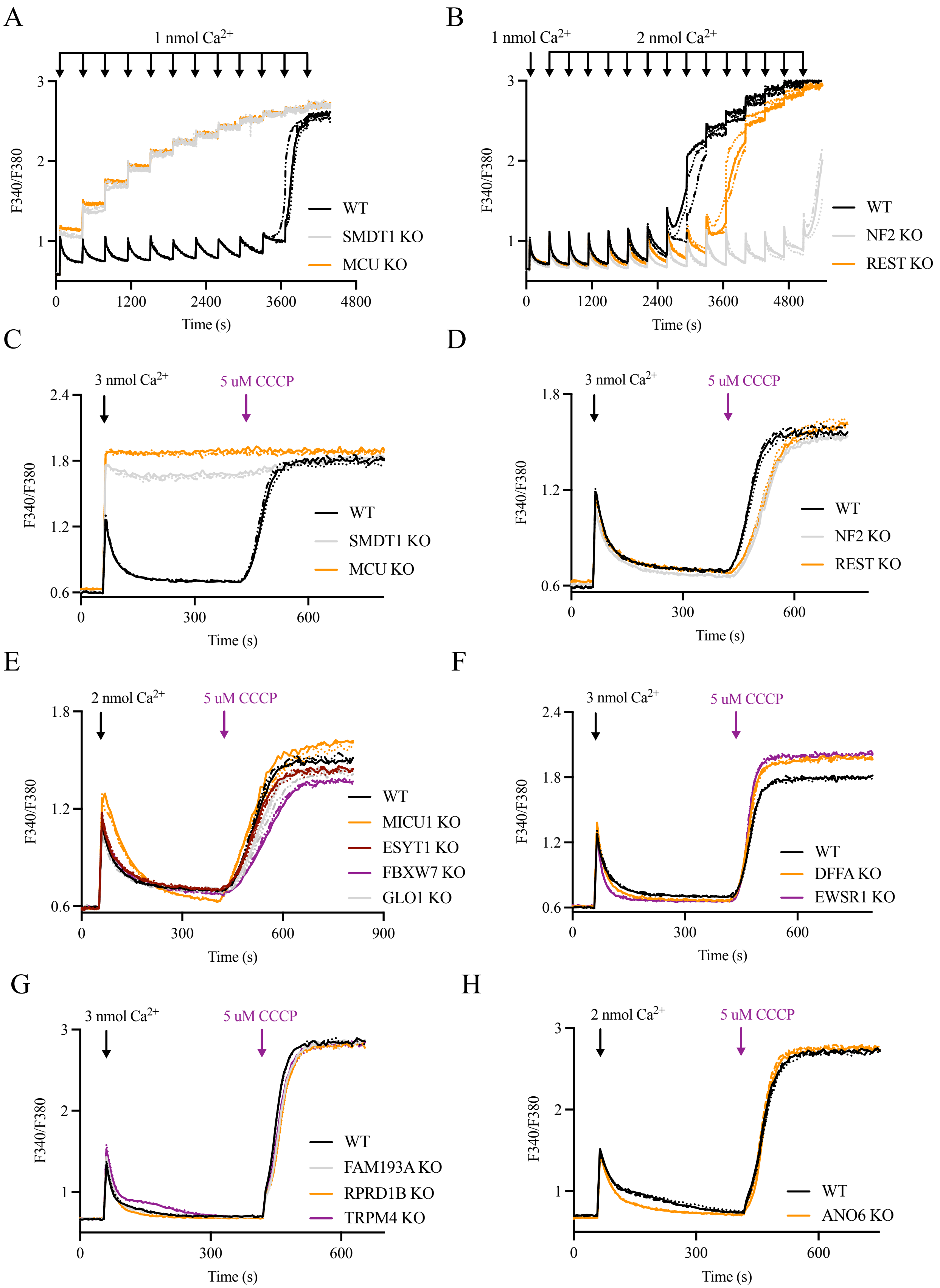
A
1 nmol Ca2+
F340/F380
Time (s)
WT
SMDT1 KO
MCU KO
B
1 nmol Ca2+
2 nmol Ca2+
F340/F380
Time (s)
WT
NF2 KO
REST KO
C
3 nmol Ca2+
5 uM CCCP
F340/F380
Time (s)
WT
SMDT1 KO
MCU KO
D
3 nmol Ca2+
5 uM CCCP
F340/F380
Time (s)
WT
NF2 KO
REST KO
E
2 nmol Ca2+
5 uM CCCP
F340/F380
Time (s)
WT
MICU1 KO
ESYT1 KO
FBXW7 KO
GLO1 KO
F
3 nmol Ca2+
5 uM CCCP
F340/F380
Time (s)
WT
DFFA KO
EWSR1 KO
G
3 nmol Ca2+
5 uM CCCP
F340/F380
Time (s)
WT
FAM193A KO
RPRD1B KO
TRPM4 KO
H
2 nmol Ca2+
5 uM CCCP
F340/F380
Time (s)
WT
ANO6 KO

Fig. S6. No gene knockout prevents Ca^{2+} release, but several modify Ca^{2+} uptake. (A) Representative CRC traces of SMDT1 KO (gray), MCU KO (orange), and paired control (black) in technical triplicate (n = 3). (B) Representative CRC traces of NF2 KO (gray), REST KO (orange), and paired control (black) in technical triplicate (n = 3). (C) Representative UCR traces for SMDT1 KO (gray), MCU KO (orange), and paired control (black) in technical triplicate (n = 3). (D) Representative UCR traces for NF2 KO (gray), REST KO (orange), and paired control (black) in technical triplicate (n = 3). (E) Representative UCR traces for MICU1 KO (orange), ESYT1 KO (dark red), FBXW7 KO (purple), GLO1 KO (gray), and paired control (black) in technical triplicate (n = 3). (F) Representative UCR traces for DFFA KO (orange), EWSR1 KO (purple), and paired control (black) in technical triplicate (n = 3). (G) Representative UCR traces for FAM193A KO (gray), RPRD1B KO (orange), TRPM4 KO (purple), and paired control (black) in technical duplicate (n = 2). (H) Representative UCR traces for ANO6 KO (orange) and paired control (black) in technical triplicate (n = 3). In all plots, digitonin-permeabilized HAP1 cells were seeded at 2 million cells/well in a 96-well blackout plate for Ca^{2+} imaging (1 μM Fura-FF; F340/F380). For CRC (A and B), Ca^{2+} boluses were applied until large-scale Ca^{2+} release occurred. For UCR (C–H), a single 2 or 3 nmol Ca^{2+} bolus was added prior to an uncoupler CCCP (5 μM final), and fluorescence recorded until Ca^{2+} release was complete.

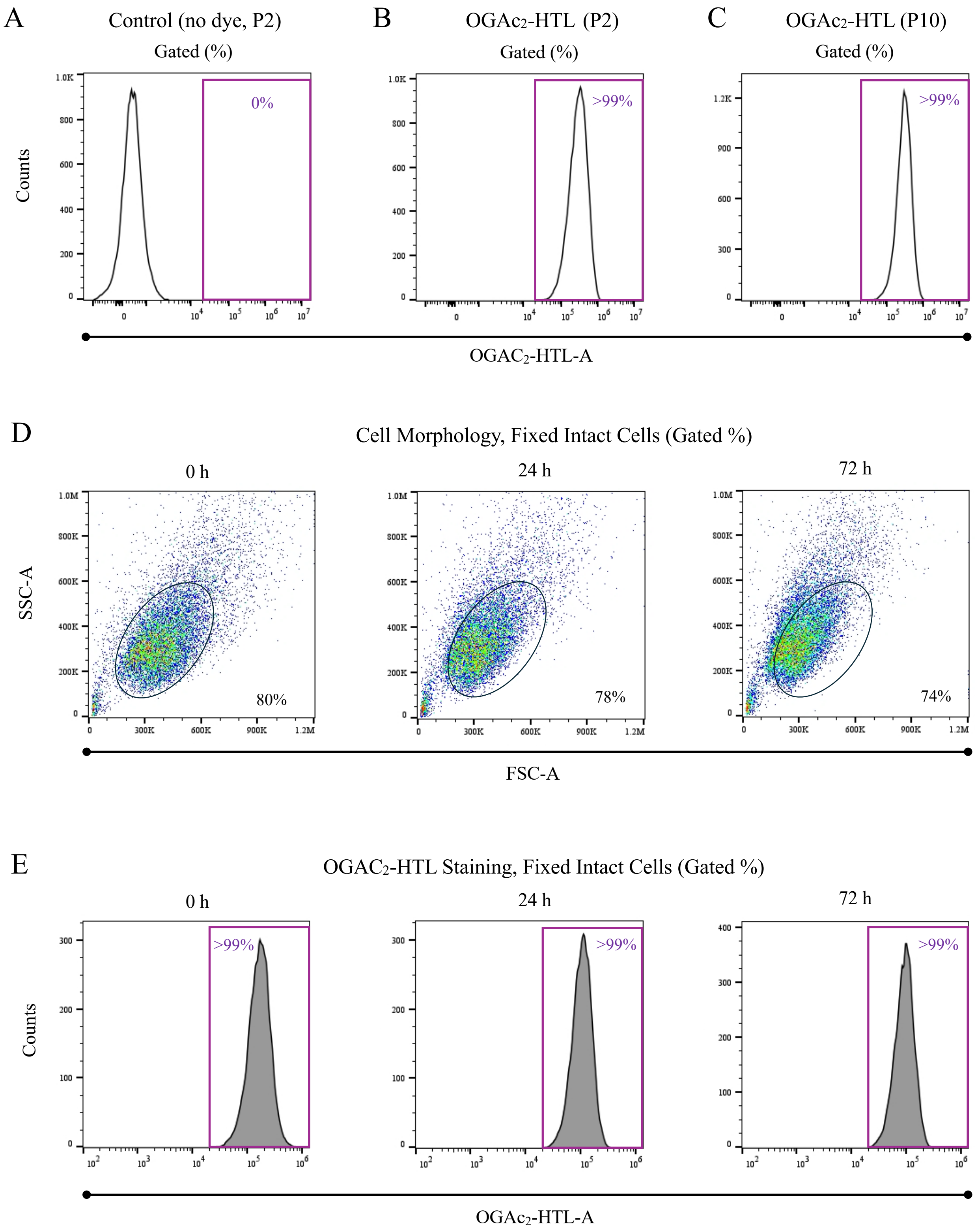
A
Control (no dye, P2)
Gated (%)
0%
B
OGAc2-HTL (P2)
Gated (%)
>99%
C
OGAc2-HTL (P10)
Gated (%)
>99%
Counts
OGAC2-HTL-A
D
Cell Morphology, Fixed Intact Cells (Gated %)
0 h
24 h
72 h
SSC-A
80%
78%
74%
FSC-A
E
OGAC2-HTL Staining, Fixed Intact Cells (Gated %)
0 h
24 h
72 h
Counts
>99%
>99%
>99%
OGAc2-HTL-A

Fig. S7. Development and optimization of the mitochondrial permeability assay. (A – C) HAP1SR-HaloMTS cells maintain HaloMTS expression for at least 8 passages. Flow cytometry histograms of intact HAP1SR-HaloMTS cells in control (no dye, P2; A) and 30 nM acetylated Oregon Green HaloTag ligand ($OGAC_2$-HTL; B and C); immediately after generation (P2; A and B) or P10 (C). (D) Cell size (FSC-A) declines each day after fixation with intact HAP1SR-HaloMTS cells. Flow cytometry plots for cell morphology (SSC-A vs. FSC-A) at 0 h, 24 h, and 72 h after fixation with 4% PFA (30 min, ice). The gated population in all plots corresponds to the day 0 population. Percentages reflect population totals within the gate. (E) $OGAC_2$-HTL staining is mostly unaltered by fixation for at least 72 h. Flow cytometry histograms of intact HAP1SR-HaloMTS cells stained with 30 nM $OGAC_2$-HTL and recorded at 0 h (left), 24 h (middle), and 72 h (right) post fixation (4% PFA, 30 min, ice). In (A – C and E), particles were first gated for cell morphology (SSC-A vs. FSC-A), followed by singlets (FSC-A vs. FSC-W), and then $OGAC_2$-HTL fluorescence ($OGAC_2$-HTL-A). The gated population reflects HaloMTS expression (A) and $OGAC_2$-HTL persistence (E); percentages are the expressing population.

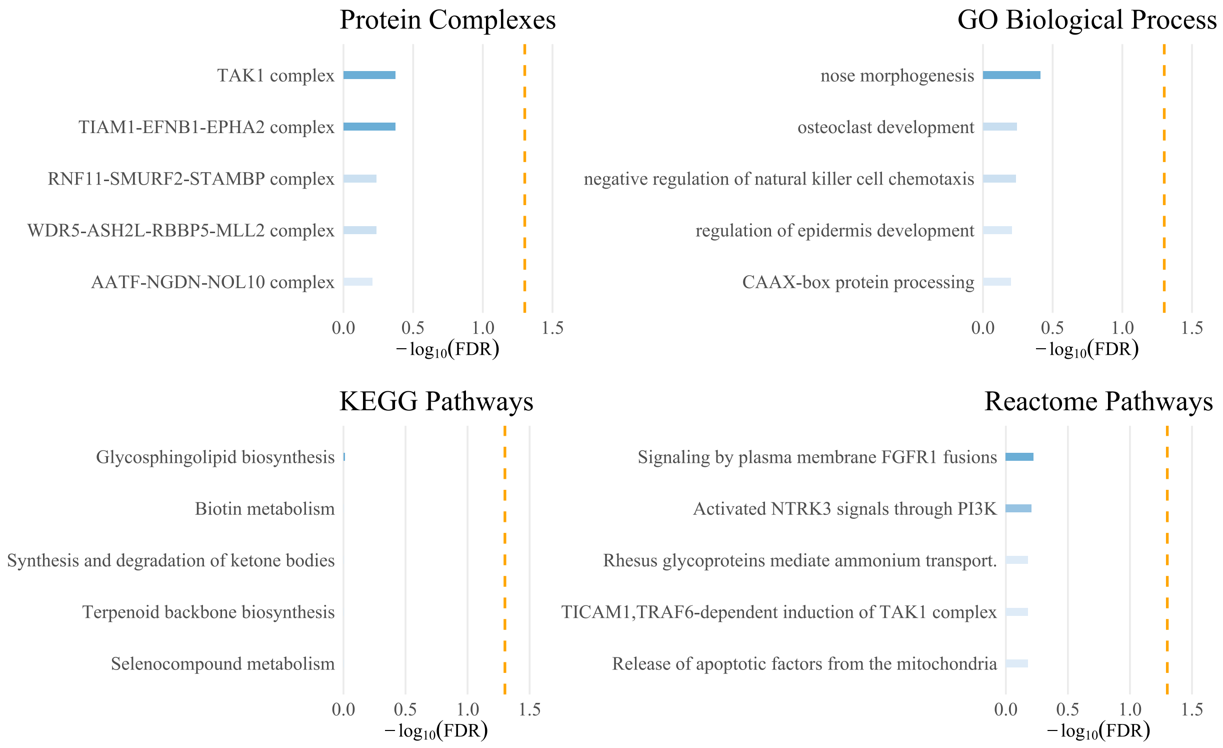

Fig. S8. Gene set enrichment analysis (GSEA) for the mitochondrial permeability CRISPR screen. Statistically significant enriched pathways were not found. GSEA was performed on genes ranked by positive RRA, and enrichment was tested across Protein Complexes, GO Biological Processes, KEGG, and Reactome Pathways. The top five pathways plotted by -$\log_{10}$(FDR). Statistical significance (-$\log_{10}$(FDR) = 1.3; FDR < 0.05) is denoted by the dashed orange line.

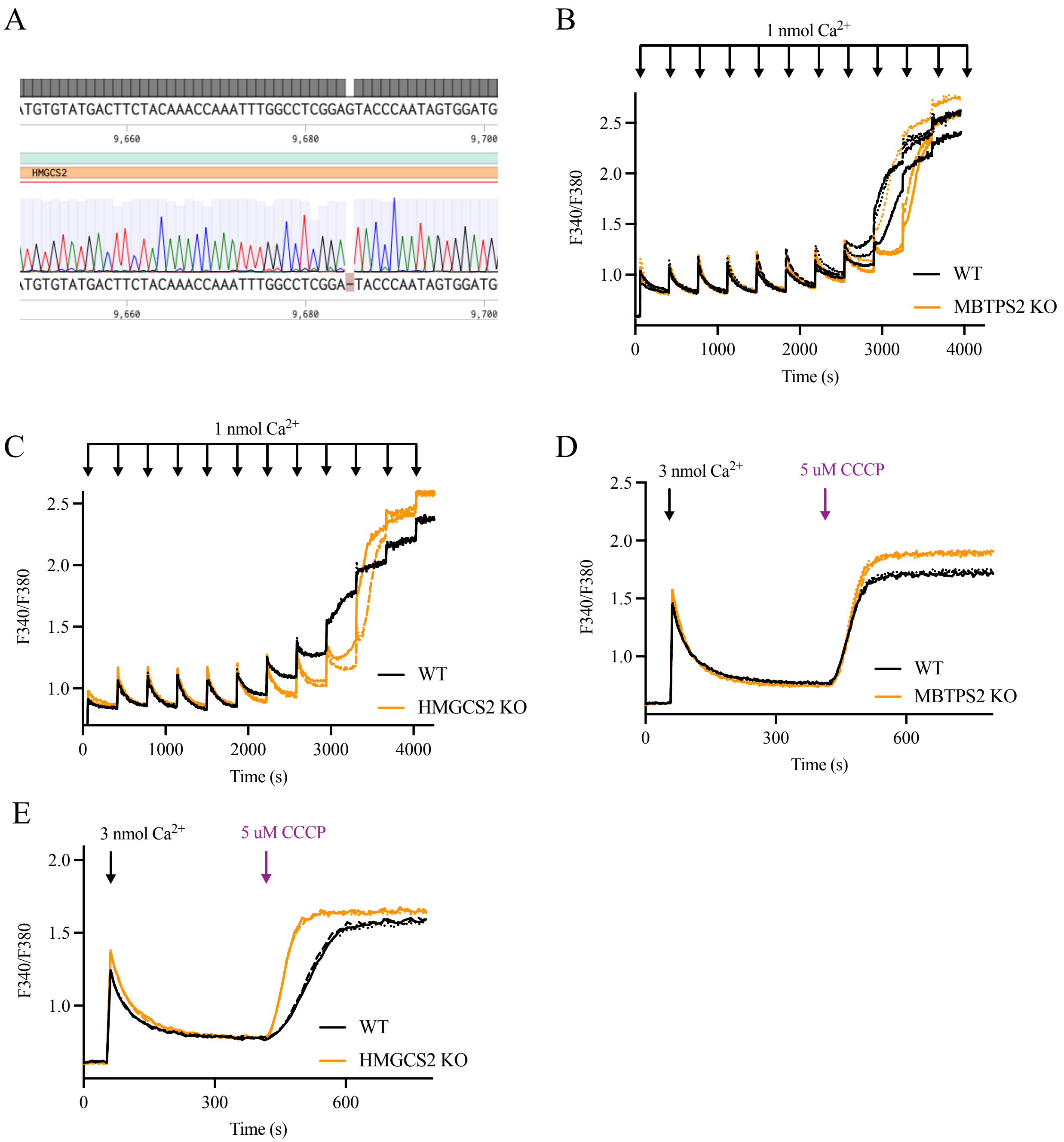

Fig. S9. Knockout of MBTPS2 or HMGCS2 does not prevent Ca^{2+} release. (A) Sanger sequencing chromatogram aligned with the HMGCS2 *Homo sapiens* gene in Benchling. From ~50,000 clonal cells, gDNA was isolated, sgRNA containing amplicons were PCR amplified, purified by column filtration, and sequenced by Sanger sequencing to identify a frameshift indel. MBTPS2 KO or HMGCS2 KO do not impact CRC or prevent Ca^{2+} release. Representative CRC traces for MBTPS2 KO (B) and HMGCS2 KO (C) comparing WT (black) and KO (orange) cells. In each, 1 nmol Ca^{2+} boluses were added until large-scale Ca^{2+} release occurred. MBTPS2 KO or HMGCS2 KO do not prevent the UCR. Representative UCR traces for MBTPS2 KO (D) and HMGCS2 KO (E) comparing WT (black) and KO (orange) cells. In each, a 3 nmol Ca^{2+}

bolus was added prior to an uncoupler (5 μM CCCP), and fluorescence was recorded until Ca^{2+} release was complete. (B – E) Digitonin-permeabilized HAP1 cells were seeded at 2 million cells/well in a 96-well blackout plate for Ca^{2+} imaging (1 μM Fura-FF; F340/F380).

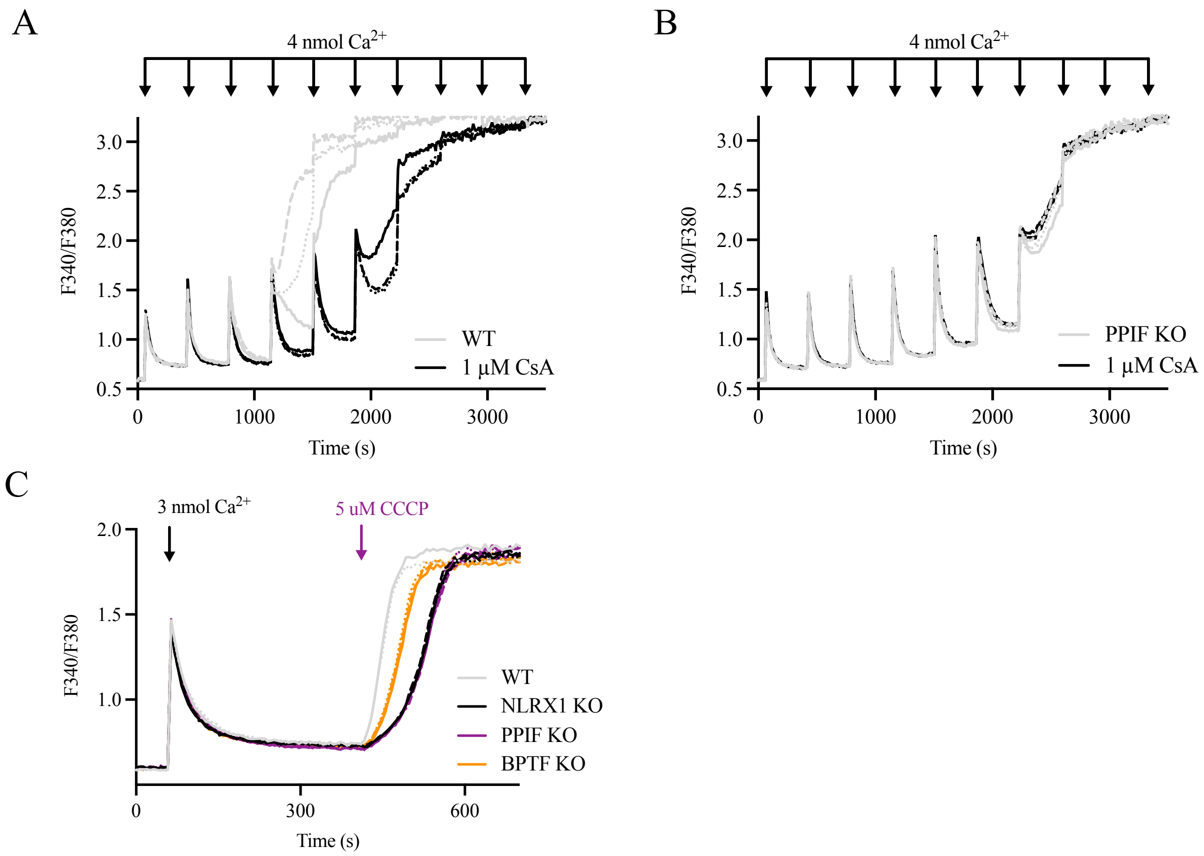

Fig. S10. CRC controls for CsA experiments and UCR for the top three hits of the mitochondrial permeability CRISPR screen. (A and B) CRC comparing control (gray) and 1 µM CsA (black) in WT (A) and PPIF KO (B) with 4 nmol Ca^{2+} boluses until large-scale Ca^{2+} release occurred. (C) No candidate KO prevents UCR. WT (gray), NLRX1 KO (black), PPIF KO (purple), and BPTF KO (orange) cells were given a 3 nmol Ca^{2+} bolus prior to the uncoupler (5 µM CCCP), and fluorescence was recorded until Ca^{2+} release was complete. For all plots, digitonin-permeabilized HAP1 cells were seeded at 2 million cells/well in a 96-well blackout plate for Ca^{2+} imaging (1 µM Fura-FF; F340/F380).

Dataset S1. MAGeCK next generation sequencing analysis for the genome-wide mitochondrial membrane potential FACS screen.

Dataset S2. MAGeCK next generation sequencing analysis for the genome-wide mitochondrial permeability FACS screen.
